# Nested plant LTR retrotransposons target specific regions of other elements, while all LTR retrotransposons often target palindromes and nucleosome-occupied regions: in silico study

**DOI:** 10.1186/s13100-019-0186-z

**Published:** 2019-12-14

**Authors:** Pavel Jedlicka, Matej Lexa, Ivan Vanat, Roman Hobza, Eduard Kejnovsky

**Affiliations:** 1 Department of Plant Developmental Genetics, Institute of Biophysics of the Czech Academy of Sciences, Kralovopolska 135, 61200 Brno, Czech Republic; 2 Faculty of Informatics, Masaryk University, Botanicka 68a, 60200 Brno, Czech Republic

**Keywords:** Transposable elements, LTR retrotransposons, Nesting, Chromatin, Nucleosomes, Plants

## Abstract

**Background:**

Nesting is common in LTR retrotransposons, especially in large genomes containing a high number of elements.

**Results:**

We analyzed 12 plant genomes and obtained 1491 pairs of nested and original (pre-existing) LTR retrotransposons. We systematically analyzed mutual nesting of individual LTR retrotransposons and found that certain families, more often belonging to the Ty3/gypsy than Ty1/copia superfamilies, showed a higher nesting frequency as well as a higher preference for older copies of the same family (“autoinsertions”). Nested LTR retrotransposons were preferentially located in the 3’UTR of other LTR retrotransposons, while coding and regulatory regions (LTRs) are not commonly targeted. Insertions displayed a weak preference for palindromes and were associated with a strong positional pattern of higher predicted nucleosome occupancy. Deviation from randomness in target site choice was also found in 13,983 non-nested plant LTR retrotransposons.

**Conclusions:**

We reveal that nesting of LTR retrotransposons is not random. Integration is correlated with sequence composition, secondary structure and the chromatin environment. Insertion into retrotransposon positions with a low negative impact on family fitness supports the concept of the genome being viewed as an ecosystem of various elements.

## Background

Transposable elements (TEs) are prolific structural and functional genome components colonizing genomes throughout the whole course of evolution. The plethora of types, mechanisms, modes and rates of spreading, as well as examples of domestication, all underline the importance of TEs for genome rearrangements and cell functioning [1]. Transposable elements are source of mutations, can create genes and RNAs, are epigenetically regulated and can be activated by stress as is especially evident in sessile plants where TEs represent up to 80% of the genome [2, 3].

Long terminal repeat (LTR) retrotransposons are the most common TE type in the majority of plants. Increasing accumulation of genomic sequence data during last decade enabled identification of new LTR retrotransposons. However, their classification is still being constructed. As with any taxonomic framework, the LTR retrotransposon classification system underwent revisions as diverse elements were identified. Wicker et al. [4, 5] classified Ty1/copia elements into six lineages - Maximus, Ivana, Ale, Angela, TAR and Bianca. Later, Llorens et al. [6, 7] established two lineages belonging to the Ty3/gypsy superfamily - chromovirus (composed of the Del, Reina, CRM and Galadriel clades) and Tat/Athila, and five lineages belonging to the Ty1/copia superfamily - Oryco, Sire, Retrofit, Osser and Tork. Recently, Neumann et al. [8] performed a survey of 13,863 LTR retrotransposons from 80 plant species and established a refined classification system applicable to LTR retrotransposons in plants. They divided the Ty3/gypsy and Ty1/copia elements into 14 and 16 lineages, respectively.

The genomic distribution of transposable elements differs among TE types. At least in plants, Ty3/gypsy elements are much more frequent in heterochromatin (often in centromeres) and show an opposite distribution with respect to genes, whereas Ty1/copia elements in general have a much less skewed distribution. This is the case for example of tomato [9], Arabidopsis [10], wheat [11], barley [12], sorghum [13], or soybean [14].

Some other elements only occur in specific genomic loci - e.g. miniature inverted-repeat transposable elements (MITEs) are preferentially found in gene-rich regions, or close to genes [15], Ty5 retrotransposons are integrated into telomeric heterochromatin [16], Mos1 mariner elements prefer TATA or TA motifs [17]. High TE content is characteristic for pericentromeres, TE islands and knobs that are epigenetically maintained in a silenced state forming constitutive heterochromatin (reviewed by [18]). Small genome plants like Arabidopsis have relatively few TE islands, while large genomes such as maize and wheat have greater numbers and expanded sizes of dense TE islands. Specific localization of TEs is a result of both selection processes retaining TEs only in some sites and/or targeting of elements into specific positions.

A high density of transposable elements e.g. in TE islands or knobs, can lead to TE nesting - i.e. the insertion of new elements into pre-existing elements. Extensive nesting was discovered more than two decades ago in maize [19, 20] with later studies showing TEs gathered in the centromeric regions of *Arabidopsis thaliana* [21], Drosophila [22] and Brassica [23]. Nested insertions are often biased into specific preferential positions and sequence motifs of original transposable elements as was seen in the human [24] and various eukaryotes [25]. For example, human Alu retrotransposons are more often inserted into the same type of elements and the orientation of the incorporated element is also important [24]. However, little is known about the rules governing the nesting of LTR retrotransposons that are especially abundant in plants.

Here we annotated and analyzed 1491 pairs of nested and original LTR retrotransposons from 12 plant genomes to show that nested elements are preferentially localized in specific sites of pre-existing elements.

## Results

### Nesting is more frequent in genomes with a higher density of retrotransposons

We searched 12 plant genomes (see Methods) for LTR retrotransposons nested into pre-existing LTR retrotransposons using our newly developed TE-nester tool [26, 27]. In a pair of nested elements, the younger inserted element was named “nested” and the pre-existing element was called “original”. Significance of the “younger” status was tested by comparison of average ages (insertion time in million years ago (Mya) based on LTR divergence [19, 28]) of the nested and original elements (0.99 and 2.14 Mya, respectively; two sample t-test *p* < 2.2e-16). Moreover, in order to observe a more complete picture about the LTR retrotransposons present in our target genomes, non-nested TEs were also recorded. From all analyzed genomes, we found 1491 pairs of nested and original, and 13,983 of non-nested LTR retrotransposons (Table 1). Plant species were sorted by genome size (from 119.1 to 978.5 Mbp), however this order did not fully correspond with the respective total number of TEs. The most dominant contributors to our analysis were *Sorghum bicolor*, *Physcomitrella patens* and *Glycine max* - representatives of monocots, mosses and eudicots, respectively. Altogether, these three species comprised 60.2% of all the TEs we found (Table 1).

**Table 1:**
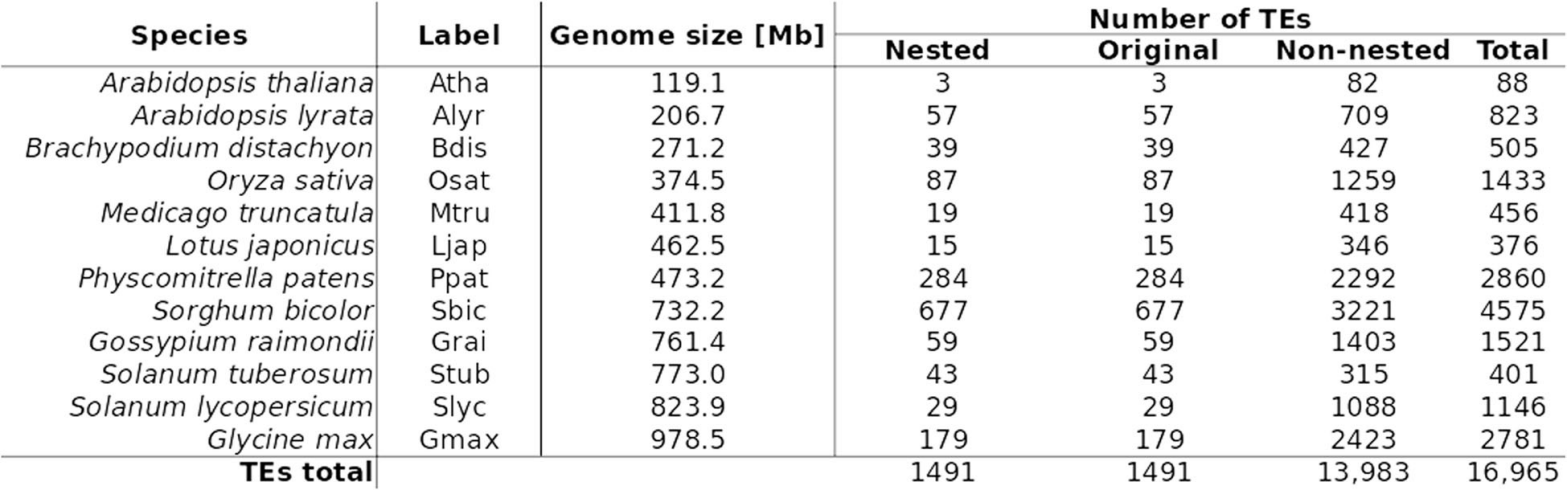
Summary table of LTR retrotransposons determined in 12 plant genomes and used for further analysis

**Fig. 1:**
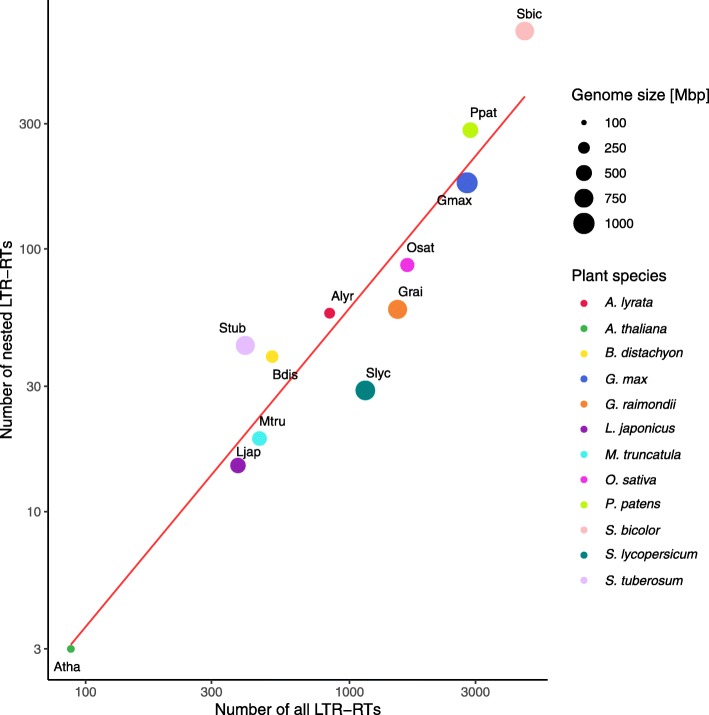
Extent of LTR retrotransposon nesting in plant genomes. Relationship between number of nested LTR retrotransposons and all LTR retrotransposons found in 12 plant genomes. Genome sizes are marked by the circle sizes. For plant species labels see Table 1

First, we calculated the extent of nesting dependent on the number of LTR retrotransposons in the particular genome. We found that nesting did not correlate with genome size but positively correlated with the number of LTR retrotransposons (Fig. 1). Analogically the lowest nesting (< 10 TEs) was detected only in *Arabidopsis thaliana* whereas the highest values (> 100 TEs) were recorded in *Sorghum bicolor*, *Physcomitrella patens* and *Glycine max*. The genome of *Sorghum bicolor* with the highest number of LTR retrotransposons had the greatest tendency for nesting. The majority of plant species showed middle tendency for nesting (10–100 TEs). Surprisingly, the closely related species of *Solanum tuberosum* and *Solanum lycopersicon* showed a similar extent of nesting despite having a different number of LTR retrotransposons (Fig. 1).

Thereafter, we quantified Ty3/gypsy and Ty1/copia representatives in the both sets of nested (2407 and 353) and non-nested (8635 and 5348) LTR retrotransposons. We showed that Ty3/gypsy superfamily is significantly overrepresented in nested LTR retrotransposons in comparison with intact TEs (Chi-square test of independence, *p* < 2.2e-16).

**Fig. 2:**
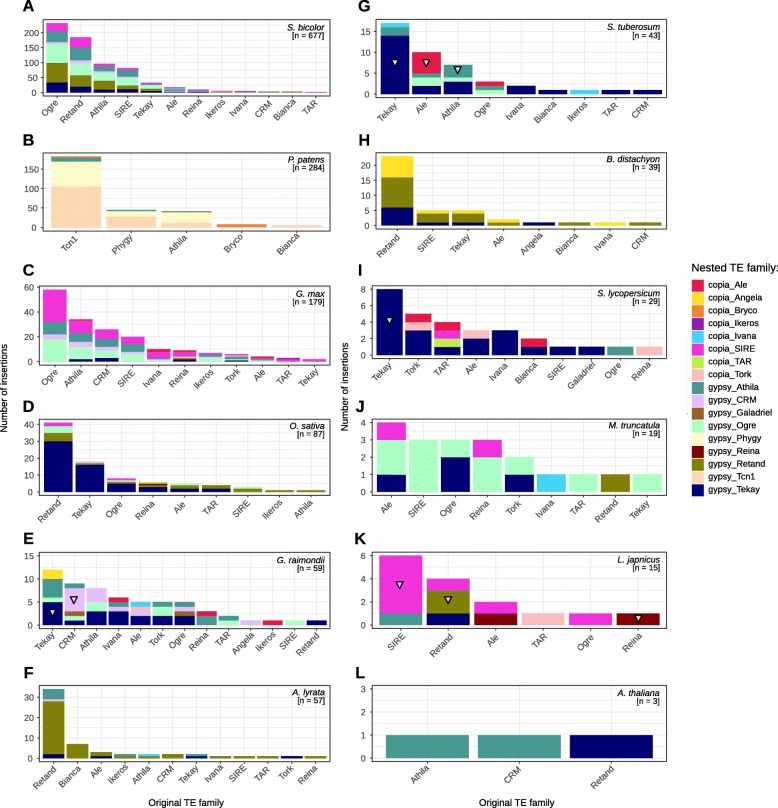
Mutual insertions of LTR retrotransposon families. Number of insertions of individual retrotransposon families (marked by different colors in vertical column) into other families (indicated below) in 12 plant species. Preferential autoinsertions (insertions into the same family) are most frequent in *Solanum tuberosum*, *Solanum lycopersicon*, *Gossyphium raimondii* and *Lotus japonicus* and are marked by triangles within columns. Plant species are ordered according to descending number of nested TEs (**a**-**l**)

Next, we looked at the localization of nested retrotransposons along their chromosomes (Fig. 2 and Additional file 1). A representative distribution pattern can be seen in the genome of *Sorghum bicolor* containing the highest number of nested-original pairs in our analysis where the extent of nesting is correlated with the density of LTR retrotransposons along chromosomes. The density of all TEs was found to be lowest in estimated pericentromeric regions e.g. chromosomes 1, 7, 9 and 10 (Additional file 1). In *Arabidopsis lyrata*, the distribution of nested elements also correlated with non-nested elements but regions of strong preferential nesting located in pericentromeres were found (Additional file 1). This phenomenon was most evident in chromosome 6. However, we should take into account that the quality of the genome assembly heavily impacts on the amount of pericentromeric sequences included in the reference genome and thus the low density of TEs found in the centromeres in *Sorghum bicolor* could be a consequence of the relative lack of centromeric sequences in the assembly compared to the very well assembled genome of *Arabidopsis lyrata*.

### Some families in some species show preferential nesting into the same family

We analyzed the mutual nesting of individual retrotransposon families in 12 plant species. In all retroelements (i.e. nested and non-nested) we identified nine Ty3/gypsy (Athila, CRM, Galadriel, Ogre, Phygy, Reina, Retand, Tcn1 and Tekay) and nine Ty1/copia families (Ale, Angela, Bryco, Bianca, Ikeros, Ivana, SIRE, TAR and Tork). Individual families nested into an original family are visualized in Fig. 2. We found that while a majority of nested LTR retrotransposons did not show any family preference, some LTR retrotransposons in some plant species were nested into the elements belonging to the same family (here we call this phenomenon “autoinsertions”), more frequently occurring in Ty3/gypsy than Ty1/copia retrotransposons. Although the highest nesting was exhibited by the most abundant families, mostly possessing long elements, autoinsertions were evident only in Tekay, Ale and Athila retrotransposons in *Solanum tuberosum*, Tekay in *Solanum lycopersicum*, Tekay and CRM in *Gossyphium raimondii*, SIRE, Retand and Reina in *Lotus japonicus* (Fig. 2).

### Preferential nesting into specific regions of LTR retrotransposons

**Fig. 3:**
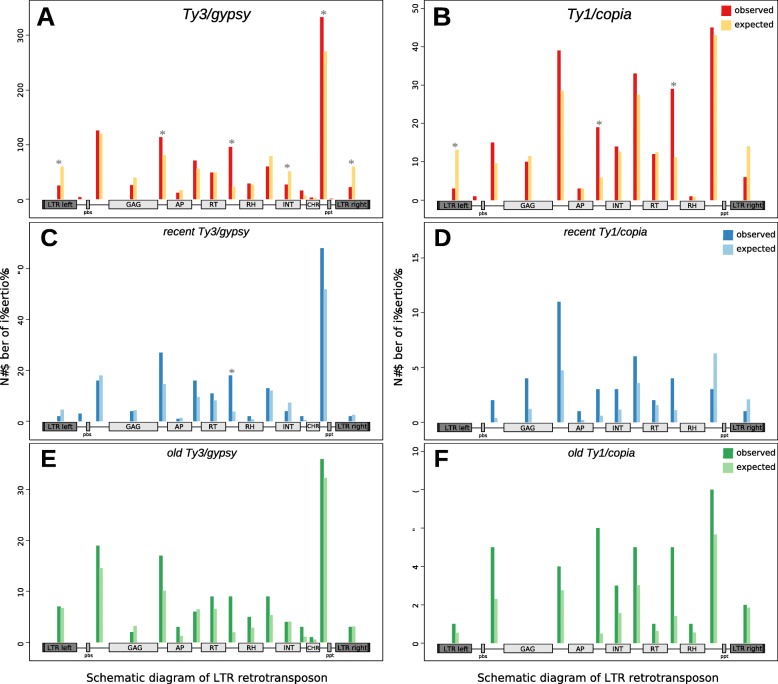
Preferential sites (hotspots) of nesting for Ty3/gypsy (**a**) and Ty1/copia (**b**) LTR retrotransposons. Bars show observed (red) number of insertions into specific retrotransposon regions together with expected (yellow) number that were normalized by the region length. The over- or under-representation of nested elements in specific regions depends on the comparison of numbers of observed and expected elements. Nesting of Ty3/gypsy (**c**, **e**) and Ty1/copia (**d**, **f**) LTR retrotransposons with high (**e**, **f**) or low (**c**, **d**) insertion time differences between original and nested insertions. Asterisks mark statistically significant differences between observed and expected numbers of nesting (*p* < 0.05; FDR corrected *p*-value of pairwise comparison after a global chi-squared goodness of fit test)

Our motivation was to find whether nesting of LTR retrotransposons is random along the original LTR retrotransposon or if any regions or motifs are preferred. Based on retrotransposon annotations, for each pair of nested and original elements we determined the site of insertion and the LTR retrotransposon region it belongs to. The regions were represented by LTRs, main protein domains (GAG, AP, RT, RH, INT, CHR), regulatory motifs (*pbs*, *ppt*) and the areas in between. The number of nesting events in these regions for Ty3/gypsy and Ty1/copia retrotransposons from all 12 plant species together is shown in Fig. 3. Due to individual LTR retrotransposons differing in length (e.g. longer 3’UTR had more insertions), the number of insertions were normalized by the average length of individual regions. The over- or under-representation of nested elements in specific regions depends on the statistical comparison of observed and expected elements (Fig. 3, Additional file 2).

We found that nested retrotransposons were not evenly distributed along pre-existing retrotransposons but were preferentially situated in specific locations (Fig. 3). They were most often present within the 3’UTR, within the region separating RT and RNAseH domains, between GAG and AP, and also within the 5’UTR. The lowest frequency of nesting was found in long terminal repeats (LTRs) and within the integrase domain. This means that insertions occupy positions in the following order (from most rare): regulatory regions (LTR), coding domains and interdomain regions.

Site preference of nested elements could be a result of either targeting into specific positions or a result of post-insertional genomic changes retaining elements in some positions while removing them from others. Both processes can be active in older elements while only targeting is detectable in younger elements. Therefore, we compared the distribution of recent and old nested elements within original TEs (pairs of elements where the period between insertion of original and nested was low or high) (Fig. 3). Our analysis of 174 old and 249 recent nested retrotransposons (having LTR identity in distance > 5% and from 0 to 1% from their original TE counterpart) showed site preference of nesting in both old and recent nested elements. This suggests that targeting as well as post-insertional changes contribute to the studied phenomenon. The more similar the preference patterns of recent and old nestings are, the more important targeting is. In Ty3/gypsy elements, the patterns of preferential nesting were similar between recent and old pairs, suggesting that Ty3/gypsy elements are often targeted to preferred positions. In Ty1/copia elements, preferential targeting was stronger in old than in recent elements, indicating that Ty1/copia accumulate in specific hotspots mostly due to post-insertional genomic changes.

Another important question was whether pairs of nested-original elements can be simultaneously mobilized. Therefore, we searched for copies of nested-original pairs with a high sequence identity (more than 80%) having the nested elements in the same or similar position within the original element. We found 26 such examples (Additional file 3A), most of them were found in *Physcomitrella patens* where Phygy elements inserted into Tcn1 were the most common. Among them, six nested elements were inserted between INT-CHR. Dot plot analysis revealed that four nested-original pairs share the same orientation while two nested-original pairs were in reverse orientation (Additional file 3B). The neighborhood of different copies of nested-original pairs was different, so we can exclude the possibility that similar copies are a result of segmental duplication and not retrotransposition. However, the insertion sites of similar copies, despite being in a similar position, always differed at least 50 bp. Therefore, we conclude that doublets of nested-original LTR retrotransposons are probably not simultaneously retrotransposed, probably due to the large size of the chimeric element, or alternatively, chimeric elements are mobilized but an additional unknown process shapes the insertion site during or after mobilization of nested structures.

### Some sequence motifs are preferred for nesting

**Fig. 4:**
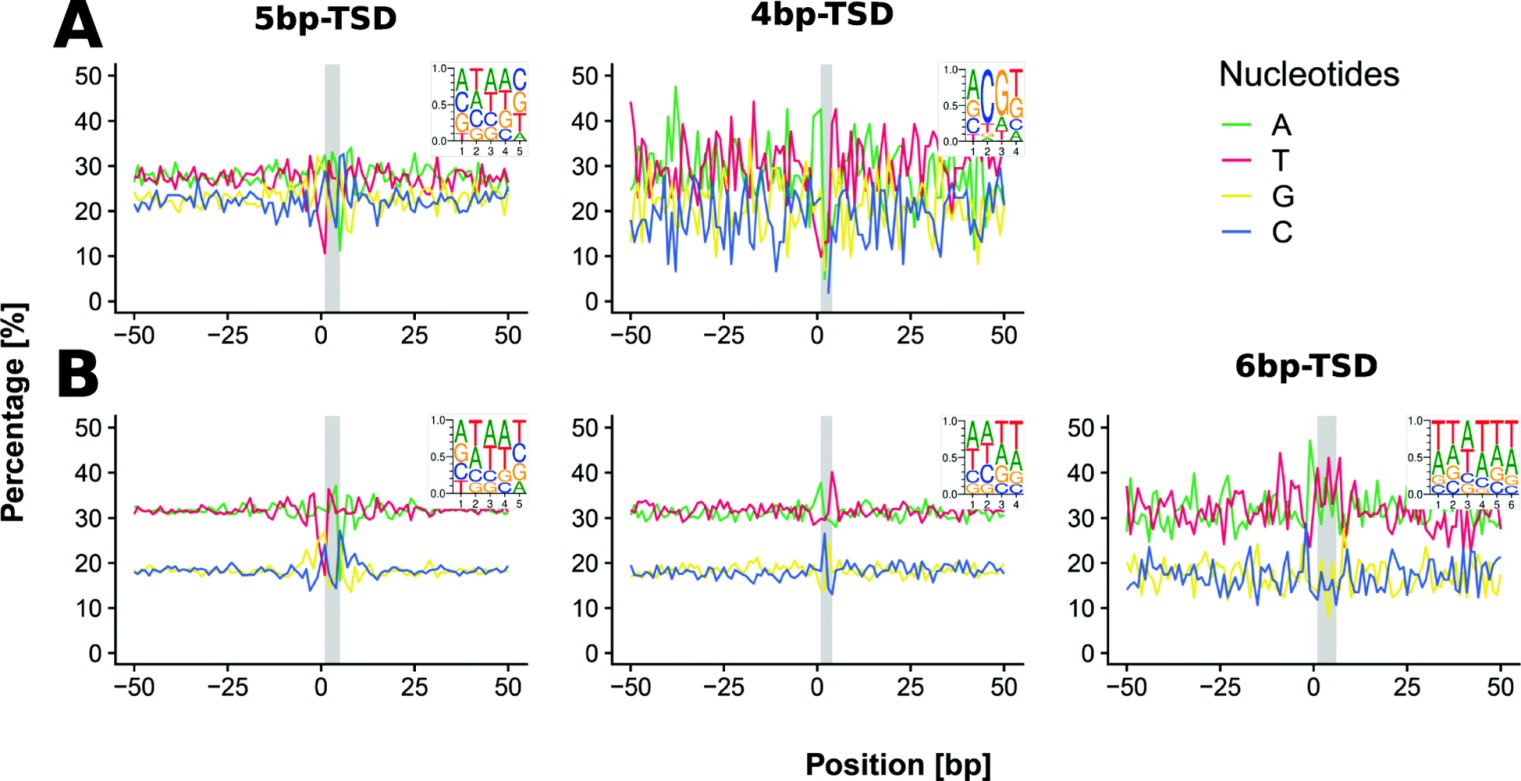
Sequence motifs surrounding nested (**a**) and non-nested (**b**) insertions. Sequence logos for LTR retrotransposons having 5bp, 4bp or 6bp target site duplications (TSD). TSD area is highlighted by vertical grey stripe. The number of insertions were as follows: 765 and 12,144 for 5bp, 61 and 1661 for 4bp TSD of nested and non-nested TEs, respectively. The 6bp TSD of non-nested TEs are represented by 178 insertions

The discovery of preferential sites of nesting motivated us to test whether there is a preferential sequence motif for targeting. Therefore, we analyzed TSD (target site duplication) and regions flanking nested elements and visualized these regions by sequence logos (Fig. 4a). We found that TSD sites (duplications formed during insertion of LTR retrotransposon cDNA by integrase) were not random and differed between common 5 bp TSDs and less abundant 4 bp and 6 bp TSDs. The 5 bp TSDs had AT rich trinucleotides in their centres flanked by A, C or G nucleotides on the left side and C, G and T on the right. T and A are strongly under-represented on the left and right side of the flanking sequence, respectively. The 4 bp TSDs had GC rich dinucleotides in the centre surrounded by AT rich nucleotides.

To obtain a complete picture of insertion site sequence composition we also analyzed non-nested LTR retrotransposons from the same 12 plant species. This analysis (where we also included 6 bp TSDs) showed similar sequence motifs (Fig. 4b). In agreement with Neumann et al. [8], the majority of both the nested and non-nested TEs were flanked by 5 bp long TSDs (in total 87.2%, *n* = 12,909), 11.6% (*n* = 1722) of TEs were flanked by 4 bp long TSDs and negligible part by 6 TSDs (1.2%, *n* = 182). The sequence surrounding the TSD had a higher GC content (45%) in nested elements compared to that of non-nested elements (38%). Indeed, a more in-depth analysis showed that LTR retrotransposons have 3–8% higher GC content compared to the entire genomes of some plant species (Additional file 4).

Moreover, both dominant types of TSD showed a palindromic character of motif. As palindromes are known to be targets of some LTR retrotransposons in certain species [29], we investigated whether there might be a longer palindrome present at the retroelement insertion site, within a 20 nucleotide base pair window. The sequences of 830 nested and 13,983 non-nested LTR retrotransposons were extracted and analysed for approximate palindromes with the paldpl program as described in Methods. Table 2 shows that on average there were 5–6 nucleotides involved in an approximate palindrome (with a maximum of 33% error rate), not more than the 2–3 base pairs seen in the TSDs. To see if any of the observed palindrome lengths were beyond what could be expected in random (or randomized) sequences, we shuffled the bases of each analysed sequence and found the average palindrome length to decrease only by less than one base (0.1–0.3 in most families). Table 2 shows these values and the results of a paired t-test comparing the shuffled and unshuffled values. While as a whole there was a statistically significant decrease of palindrome length caused by the shuffling (*p*-value = 10–6), only four TE families contributed significantly to the differences. These were Athila, Ivana, Sire and Tcn1. Taken together, our data showed that there is a slight insertion preference of LTR retrotransposon for palindromic sequence but the small absolute value of the difference suggests that any targeting is primarily driven by mechanisms other than palindrome recognition.

**Table 2:** Palindromes within sequences flanking the insertion site. We used the paldpl program to detect approximate palindromes of at least 3 bp with no more than 30% mismatches or indels. This analysis was done in native flanking sequences identified in plant genomes and their randomized (permutated) counterparts, to control for base content effects. We carried out a paired t-test for difference in calculated stem lengths of the native and randomized palindromes

Group	Count	Palindrome length		Paired t-test *p*-value
		*native*	*random*	
ALL	14,813	5.5	5.4	0.000004***
*nested*	*830*	*5.2*	*5.3*	*0.50~*
*non-nested*	*13,983*	*5.5*	*5.4*	0.000001***
Ale	1314	5.5	5.5	0.93
Alesia	21	5.8	5.7	0.75
Angela	91	5.3	5.3	0.93
Athila	1088	5.5	5.3	0.008**
*Bianca*	*443*	*6.0*	*6.1*	*0.97~*
*Bryco*	*29*	*5.8*	*5.9*	*0.95~*
CRM	482	5.3	5.2	0.53
Galadriel	49	5.4	5.1	0.40
Ikeros	348	5.5	5.3	0.10
Ivana	1018	5.5	5.3	0.008**
Ogre	1520	5.5	5.4	0.64
Phygy	285	5.3	5.3	0.94
Reina	852	5.4	5.4	0.67
Retand	2078	5.4	5.3	0.37
Sire	1225	5.4	5.2	0.001*
Tcn1	1947	5.5	5.4	0.001*
TAR	477	5.5	5.4	0.14
Tekay	1029	5.4	5.4	**0.61**
*Tork*	*517*	*5.6*	*5.8*	***0.05~***

Significant values after Benjamni-Hochberg correction for multiple family testing are marked with an asterisk and printed in bold (* for *p* < 0.2, ** for *p* < 0.05 and *** for *p* < 0.01). Four families with increased mean palindrome stem length after randomization are marked with a tilde

**Fig. 5:**
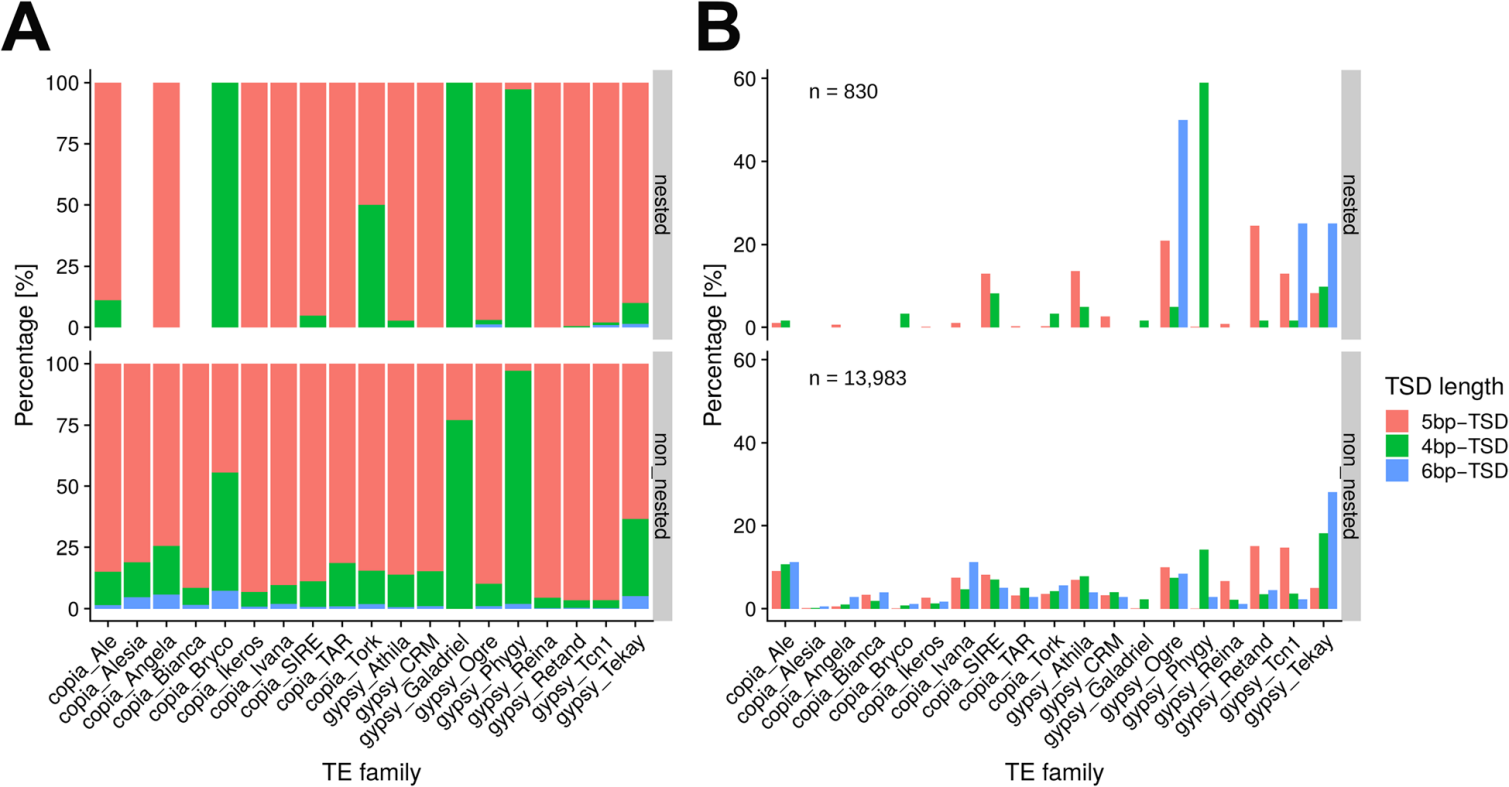
Distribution of 5 bp, 4 bp and 6 bp TSDs in retrotransposon families. **a** Proportion of 5 bp, 4 bp and 6 bp TSDs in individual LTR retrotransposon families in nested and non-nested elements. **b** Contribution of individual LTR retrotransposon families to the three main types of TSDs (5 bp, 4 bp and 6 bp)

We measured the proportions of 4 bp, 5 bp and 6 bp TSDs in the main LTR retrotransposon families and found that 5 bp TSD is the most dominant in the majority of families, forming about 80% of all TSDs. The next most abundant is 4 bp TSD, with 6 bp TSD representing only a minor proportion (Fig. 5a). Two or three TSD types could coexist in the same family. The proportion of different TSD is the same between nested and non-nested elements. Galadriel, Phygy and Bryco demonstrated a different pattern where 4 bp TSD was most common. Figure 5b shows the contribution of individual families to specific types of TSDs indicating that most 5 bp TSDs can be attributed to SIRE, Athila, Ogre, Retand, Tcn1 and Tekay while 4 bp TSDs were often represented by SIRE, Phygy, Ogre and Tekay. Finally, the majority of 6 bp TSDs can be attributed to Ogre, Tcn1 and Tekay.

### Correlation of LTR retrotransposon insertions with nucleosome positioning and occupancy

In response to the preferential autoinsertions and insertions into specific retroelement regions reported above, we examined predicted nucleosome positioning as another factor that could influence nesting in addition to insertion site preference in general. It has been previously reported in retroviruses that integrase preferentially recognizes DNA at sites occupied by a nucleosome [30]. Using the Markov Model calculations from [31] and all TEs detected by TE-nester and filtered for TSD presence (see Methods section), we analyzed 1124 bp sequence regions centered around all detected insertion sites as well as recording the predicted nucleosome occupancy at each base of the sequence.

**Fig. 6:**
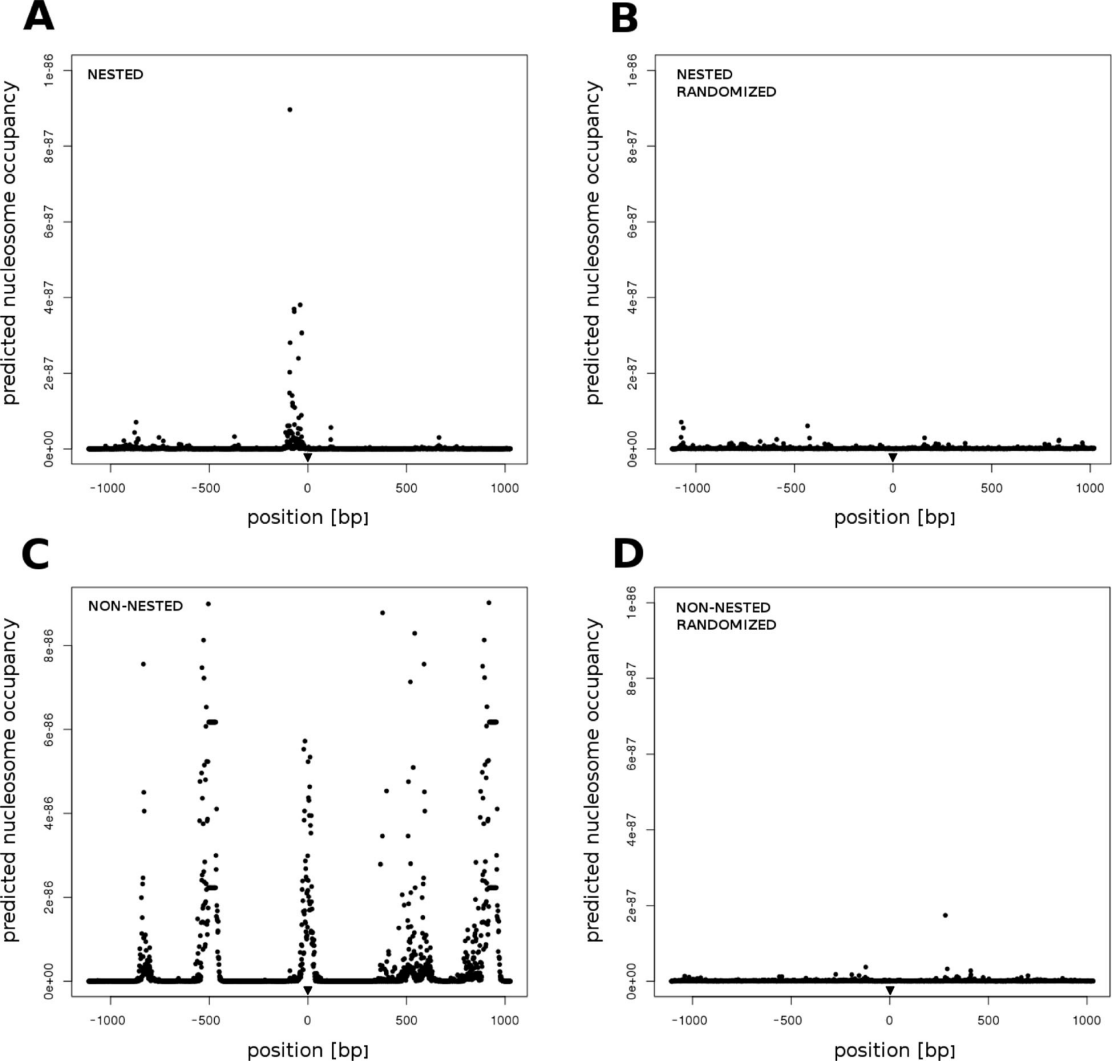
Average predicted nucleosome positioning and occupancy centered around TE insertion sites. Average predicted nucleosome occupancy at a given position near the TE insertion site (triangle) in nested (**a** - top left) and non-nested (**c** - bottom left) 1124 bp insertion site neighborhoods. Randomized sequences were analyzed using the same procedure as controls (**b**, **d** - top and bottom right). Sequences surrounding the identified insertion sites were analysed using a Markov model from Tompitak et al., 2017

This analysis revealed symmetrical patterns in the vicinity of element insertion sites identified by TE-nester, in comparison with absence of such signal in randomized DNA sequences. Figure 6a (nested) and 6C (non-nested) show the positioning predicted to place the nucleosome either near the insertion site or a few dozens bp preceding it. This is particularly apparent in the non-nested panel (Fig. 6c), probably because of the much higher number of observations. The predicted nucleosome signal is somewhat symmetrical around the insertion site, with further nucleosome positioning peaks located about 500 bp and 900 bp at both sides of the insertion site. No such phased signal was observed in nested elements, either because of the low number of observations or due to constraints placed upon the site by the original TE sequence. Sequences randomized using shuffleseq from EMBOSS did not contain any signal of comparable strength (Fig. 6b and d).

To examine a possible role of nucleosomes in regional preferences of nested insertions we also analyzed the sequences of the original TEs found by TE-nester. Separate analyses of Ty3/gypsy and Ty1/copia TEs showed consistent nucleosome positioning in the Ty3/gypsy 3′ region and similar but lower scoring regions in the Ty1/copia, including one additional group of positions in the 5’LTR (Additional file 5). This correlates with the 4.3x higher insertion rates of all nested TEs into Ty3/gypsy members than into Ty1/copia which was subsequently counted for this data set (674 and 156, respectively).

## Discussion

We found that (i) Ty3/gypsy representatives are predominant among nested LTR retrotransposons (ii) nested elements mostly do not show family preference although some families (more frequently Ty3/gypsy than Ty1/copia retrotransposons) in some plant species are preferentially nested into the same family (autoinsertions), (iii) there are preferred retrotransposon regions for nesting, and (iv) palindromic sequence motifs and nucleosome-bound regions are more often targeted than other motifs.

It is a question what factors contribute to slight over-representation of autoinertions in some families. A higher frequency of nesting into some families and sequence motifs was shown by [32]. Although the mechanisms of target site selection by retrotransposon integrase are not clear yet, we can expect that not only DNA sequence but also chromatin status and time are important here. Individual families often occupy specific genomic niches (e.g. centromeric LTR retrotransposons). Such behavior increases the probability of autoinsertion. Also, expansion waves have the potential to enrich the genome for young copies belonging to the expanding families. In contrast, genomic competition of retrotransposon families - results in the suppression of autoinsertions. Namely, if the genome is viewed as an ecosystem of elements competing for individual genomic loci [33], disruption of the elements belonging to the same family would be disadvantageous for any active family.

The nesting of elements into non-genic positions (e.g. 3’UTRs) as such as we found, is advantageous for any family because such insertions, if elements are still transcribed, do not reduce the protein pool of a specific family and thus are less detrimental. On the other hand, insertions into genic (RT or INT domains) and regulatory regions (LTRs) were suppressed similarly as was observed by [34]. Insertions into regulatory regions like TATA boxes, *cis*-acting elements and repetitive regions within LTRs are probably the most damaging because LTR retrotransposons can not jump with damaged LTR but when e.g. the pol gene is damaged, they can borrow enzymes from other family members (from the cellular pool). At this time we are not however able to explain the observed frequent insertions into regions between GAG and POL genes.

Despite a high proportion of insertions probably being random, our finding of site preference during the nesting of retrotransposons, where the period between the insertion of the original and nested was short, demonstrates that targeting is an important factor. Although the targeting was also supported by an identification of preferred sequence motifs composing TSD, we are aware that recognition of specific retrotransposon regions and specific sequence motifs can be mediated by various mechanisms. Thus, our data support the view that both targeting into specific regions and random insertions take place during nesting.

The target site preference of plant LTR retrotransposons has been previously demonstrated in the rice retrotransposon Tos17 where it recognizes palindromic consensus sequence ANGTT-TSD-AACNT, flanking the 5 bp TSD [29]. Selection of palindromes can reflect the multimerization of integrase within the integration complex. Apart from plants, deviation from randomness has also been shown in target sequences of yeast Ty1 retrotransposons [35]. Moreover, palindromes have preference in DNA transposon as was shown e.g. in drosophila [36, 37]. Our analysis of thousands of nested and non-nested TSDs of LTR retrotransposons in 12 plant species showed that the 5 bp, 4 bp and 6 bp TSDs differed in sequence composition (CG-rich in the outer or inner part, respectively) and furthermore, that different TE families prefered TSDs of specific sizes, indicating a different mode of recognition by integrase. Moreover, the TSD vicinities of both nested and non-nested TEs showed separate GC content patterns. Thus, this feature is not a primary factor for target preference such as that seen in Tos17 Ty1/copia TE in rice [29].

The AA dinucleotide found in 5 bp TSD has the highest propensity for DNA bending. The wedge model for DNA bending assumes that the AA dinucleotide contains a high “wedge” angle that causes a deflection in the axis of the DNA double helix [38]. Therefore, it is possible that this changed DNA conformation is recognized by integrase when central nucleotides are bent and DNA grooves are widened which allow cleavage [39]. Moreover, palindromes in TSDs have a higher tendency for the formation of hairpins representing another deviation from canonical DNA conformation. Moreover, our analysis showed that such conclusions are not confined to nested LTR retrotransposons but are also valid for other (non-nested) LTR retrotransposons.

Our finding that TE insertion sites have a non-random distribution of computationally predicted nucleosome positions and occupancy raises several interesting questions. Both nested and non-nested insertion sites showed increased nucleosome predictions in the immediate neighborhood of the insertions (Fig. 6), which corresponds to similar findings in retroviruses [30]. Moreover, non-nested insertion sites contained additional signals on both sides of the insertion site (Fig. 6c). This finding corresponds with rather irregular nucleosome density described in *Saccharomyces cerevisiae* [40]. Similar analyses of LTR retrotransposon sequences only showed a stronger signal in the Ty3/gypsy 3’UTR region where we observed an increased insertion rate compared to other retrotransposon regions. On the other hand, the special chromatin environment of the LTRs (containing retrotransposon transcription start sites) could have affected the neighboring sequences and their nucleosome phasing and occupancy, perhaps as suggested by [41] or [42]. Therefore, we can not exclude the possibility that nucleosome positioning was the consequence and not the cause of the insertions.

Retrotransposon nesting can also fulfil a regulatory role. Since nesting often results in the inactivation of the pre-existing retrotransposon, such a process represents a mechanism of self-regulation reducing the harmful effect of the retrotransposon on the host. Some retrotransposons, in particular gypsy elements, spread heterochromatic marks into neighbouring genes to regulate their expression [43]. In nested elements, epigenetic marks can be spread from both sides (from pre-existing elements) compared to side by side insertions. In this way, a greater amount of nesting can contribute to silencing and the subsequent spreading of the silencing signal along chromosomes and the heterochromatinization of retrotransposon-rich regions. Moreover, some retrotransposons preferentially target heterochromatin [32, 44].

LTR retrotransposons are often gathered in plant centromeres [45, 46] or in heterochromatin knobs in maize [47]. The discovery of trans-chromosome TE interactions, termed KNOT [48], opened a question whether such regions could represent preferred landing sites for transposable elements and thus contribute to TE regulation. In this way, through chromatin changes, nesting could contribute to the regulation of both retrotransposon proliferation and also to the opposing process of ectopic recombination. Genome expansion and contraction is balanced and contributes to the higher genome dynamics of plants compared to animals [49].

Moreover, the nesting of LTR retrotransposons has a practical use in evolutionary studies as a unique tool for the estimation of LTR retrotransposon relative age – the nested element is always younger than the pre-existing element. Gene conversion has probably led to the underestimation of LTR retrotransposon age in many studies using the LTR divergence method, and so this popular dating approach widely applied in plants is no longer sufficient. Thus, alternative methods (e.g. [50]) based on more approaches, including an absolute chronology obtained from mutual nesting, are needed.

## Conclusions

The contribution of targeting and randomness in LTR retrotransposon insertions is a subject of long lasting debate and so important focus of research during the last three decades. In this paper we studied a set of 1491 nested TE pairs from 12 publicly available plant genomes. The set was obtained specifically for this purpose with a newly developed program TE-nester.

We have shown that nesting of LTR retrotransposons is not entirely random in plant genomes. Ty3/gypsy exhibited a higher nesting frequency than Ty1/copia families. Preferential insertions into the same LTR retrotransposon family were observed only in dicot species and were more common in Ty3/gypsy than in Ty1/copia families. Integration of nested LTR retrotransposons is correlated with sequence composition, secondary structure (palindromes) and chromatin environment. Nested LTR retrotransposons were preferentially located in the 3’UTR of other LTR retrotransposons, while coding and regulatory regions (LTRs) were not so commonly targeted.

Insertion into positions with a low negative impact on family fitness supports the concept of the genome being viewed as an ecosystem of various elements. Deeper insight into the mechanisms of LTR retrotransposon targeting can help to understand how mobile elements can shape genome structure. Overall it should of course be noted that our general conclusions are based on only 12 sequenced genomes and the situation in other plant genomes could differ, just as the patterns of nesting (e.g. autoinsertions) among plants varied in the presented analysis.

## Methods

### Genomic sequence sources and TE annotation

All of the plant genomes covering diverse taxons of higher plants were downloaded from Phytozome 12.0 [51, 52] apart from *Lotus japonicus* [53]. The 12 species and their respective genome versions included *Arabidopsis lyrata* ([54], Alyrata_384_v1.fa), *Arabidopsis thaliana* ([55], Athaliana_167_TAIR9.fa), *Brachypodium distachyon* ([56], Bdistachyon_314_v3.0.fa), *Glycine max* ([14], Gmax_275_v2.0.fa), *Gossypium raimondii* ([57], Graimondii_221_v2.0.fa), *Lotus japonicus* ([58],, Lj2.5_genome_contigs.fna.gz), *Medicago truncatula* ([59], Mtruncatula_285_Mt4.0.fa), *Oryza sativa* ([60], Osativa_323_v7.0.fa), *Physcomitrella patens* ([61], Ppatens_318_v3.fa), *Sorghum bicolor* ([62], Sbicolor_313_v3.0.fa), *Solanum lycopersicum* ([63], Slycopersicum_390_v2.5.fa) and *Solanum tuberosum* ([64], Stuberosum_448_v4.03.fa). Unmasked sequences were analysed with TE-nester [26, 27]. TE-nester in its latest version relies upon LTR Finder [65] to identify full-length LTR retroelements. It recursively removes the identified elements from the analyzed genomes so that other full-length copies fragmented by nesting can be discovered with the same tools. The annotations were saved as GFF3 files for visualization and downstream analysis. They contained information on the positions of entire elements as well as their structural components (LTR, PBS, PPT, *gag* and *pol* gene protein domain sequences, TSD). Subsequences of interest (LTR, RT domain, insertion sites) were extracted from downloaded genome sequences using bedtools package [66]. Moreover, TE-nester also retrieves sequences of all annotated TEs in ‘fasta’ format [26].

### Qualitative selection of nested-original and non-nested LTR retrotransposon and their family determination

In order to include only reliably determined TEs in our analyses, their selection was conducted in three consecutive steps using a series of in-house scripts written in python, bash and/or perl languages. At the first selection stage, the coordinates of each transposable element (TE) and the presence of the RT domain given in GFF3 files were used as position and annotation quality criterions, respectively. Therefore TEs with boundaries present within the coordinates of another TE was simply considered as ‘nested’ and ‘original’, respectively. Vice versa, the solitary TE was labeled as ‘non-nested’. In the case of multiple level nesting (i.e. nested TE also hosted another nested TE within it, etc), only the ‘first level/floor’ of nested TEs were counted in the pair with the original TE. Furthermore, in order to determine the family of any given TE and to confirm the domain presence and position given by the TE-nester, the sequences from filtered nested pairs and non-nested TEs were re-annotated using DANTE, a TE protein domain finder, available in RepeatExplorer server [67, 68]. DANTE was chosen since this tool employs an up-to-date and comprehensive database of TE domains with their taxonomic affiliations to respective families [8]. The domain coordinates obtained from DANTE were recalculated back to the offsets in chromosomes and TEs were selected based on intersects with the positions of the corresponding domains found by TE-nester (for the python scripts used see Additional file 6). Finally, the nested-original TE pairs were filtered based on the occurrence of target site duplications (TSDs) in original (i.e. older) TEs from each pair and similarly only non-nested TEs with TSDs were used for subsequent analysis. In total we started our analysis with 1491 nested TE pairs and 13,983 non-nested TEs from 12 plant genomes which were annotated into 18 families according to Neumann et al. [8] (the fully annotated GFF3 files were compressed and are provided as Additional file 7).

### Further bioinformatic analysis

The data from pre-processed GFF3 and respective genomic and TE sequence FASTA files were analyzed using a series of custom BioPython [69] scripts and R [70] with relevant packages (‘ggplot2’: [71]; ‘gplots’: [72]) was used for their visualization. Specific requirements and process steps/pipelines for figures were as follows: Genome sizes given in Fig. 1 were calculated from each assembly used. The exon coordinates presented in Additional file 1 were taken from GFF3 files [51, 52]. The GFF3 file with exons of *L. japonicus* was downloaded from [73]. The approximate positions of the centromeric regions in *S. bicolor* and *A. lyrata* (Additional file 1) were adopted from [74, 75], respectively. The schema of Ty3/gypsy and Ty1/copia LTR retrotransposons in Fig. 3 are generalized. In cases where the original Ty3/gypsy family does not contain chromodomain, the insertions are displayed between the ‘CHR’ and ‘ppt’ segments in our visualization. Since all the obtained LTR retrotransposons did not have detected complete sets of protein domains and non-coding regions, the average lengths of all these segments were received for 14 LTR retrotransposon families and precise position of nested elements was established in 1245 original elements (1015 and 230 of Ty3/gypsy and Ty1/copia, respectively). Number of expected insertions into each segment of original retrotransposon was counted as follows: (average segment length / whole element length) * number of all nested elements in respective retrotransposon family. Resulted expected counts are visualized beside the observed numbers in Fig. 3. Relative time between the insertion of original TE into the genome and nested TE into the original one was determined as the difference between their LTR identities (LTR identity of nested minus original). Global alignment algorithm counted by ‘stretcher’ function in Emboss 6.6.0 [76] was employed for this task. Two separate groups of TE pairs labeled as ‘recent’ and ‘old’ were filtered afterwards. The ‘recent’ are pairs with LTR identity delta from 0 to 1% (*n* = 229) and the ‘old’ pairs are those with LTR identity delta equal or higher than 5% (*n* = 174). For base composition analysis of insertion ‘hotspots’ demonstrated in Figs. 4 and 5, only TE pairs in which nested TE sequences are flanked by target site duplications (TSDs) were filtered. Therefore, from our original dataset of 1491 TE pairs we obtained 765, 61 and 4 hotspots (i.e. TE pairs) with TSD lengths 5, 4 and 6 base pairs, respectively (830 TE pairs in total). The same datasets were used for palindromic sequence and nucleosome positioning and occupancy evaluations (Table 2 and Fig. 6). The flanked 50 bp long sequences surrounding the hotspots were excised using ‘getfasta’ command in ‘BEDTools’ suite, version v2.25.0 [66]. The ‘WebLogo’ tool version 3.6 was used for sequence logos presented in right upper corners in Fig. 4 [77, 78].

### Insertion site evaluation for palindromic sequence presence

Using the GFF3 annotation files we extracted 20 bp flanking regions of insertion sites, including the TSD, with 10 bp in each direction. These sequences were analysed for the presence of approximate palindromes using the paldpl program [79], requiring a minimum length of 2 × 3 = 6 bp and allowing for a maximum of 33% error rate (both mismatches and indels). The stem length of the palindrome was used as a score to evaluate the potential TE insertion preference for palindrome-containing sequences.

### Insertion site evaluation for nucleosome positioning and occupancy

To estimate nucleosome status in the vicinity of TE insertion sites, we employed the method of nucleosome positioning prediction with Markov chains [31]. Sequences in FASTA format were analysed with their nucleosome_prob_landscape.py script [80]. Randomized sequences were generated by changing the order of nucleotide bases in the FASTA files, using the EMBOSS shuffleseq application [76]. Results of the analysis were averaged over all available sequences and plotted for further investigation.

## Supplementary information

**Additional file 1.** Chromosomal localization of nested and non-nested LTR retrotransposons in all studied plant species.

**Additional file 2. **Tables of statistical support for the preferential nesting into specific regions of LTR retrotransposons (presented in Fig. 3). The number of observed LTR retrotransposon insertion was compared with their expected number normalized by region length. FDR corrected *p*-values present the results of pairwise comparison after a global chi-squared goodness of fit test. The *p*-values lower than 0.05 are in bold.

**Additional file 3. **Copies of nested-original pairs with the high sequence identity. (A) Plant species and LTR retrotransposon family affiliance. (B) Dot plots of *P. patens* Phygy-Tcn1 complexes with common insertion between INT and CHR.

**Additional file 4.** Difference in GC content between LTR retrotransposons and whole genome sequence in all plant species.

**Additional file 5.** Nucleosome occupancy prediction counted for Ty3/gypsy or Ty1/copia superfamilies members detected as nested or non-nested LTR retrotransposons in this study.

**Additional file 6.** Python scripts used for recalculation of genomic coordinates of protein domains in analyzed LTR retrotransposons annotated using DANTE tool, RepeatExplorer.

**Additional file 7.** GFF3 files of fully annotated nested-original pairs and non-nested LTR retrotransposons used in this study.

## Data Availability

The datasets generated and analyzed during our study are included as Additional files.
