# Correction to: Nested plant LTR retrotransposons target specific regions of other elements, while all LTR retrotransposons often target palindromes and nucleosome-occupied regions: in silico study

**DOI:** 10.1186/s13100-019-0199-7

**Published:** 2020-01-07

**Authors:** Pavel Jedlicka, Matej Lexa, Ivan Vanat, Roman Hobza, Eduard Kejnovsky

**Affiliations:** 1 Department of Plant Developmental Genetics, Institute of Biophysics of the Czech Academy of Sciences, Kralovopolska 135, 61200 Brno, Czech Republic; 2 Faculty of Informatics, Masaryk University, Botanicka 68a, 60200 Brno, Czech Republic

**Correction to: Mobile DNA**

**https://doi.org/10.1186/s13100-019-0186-z**

Following publication of the original article [1], the authors spotted an error in Table 2.

- t-test pvalue in penultimate row = “0.61” should not be bold
- the asterisks with significance levels should be as follows: “(* for p < 0.1, ** for p < 0.01 and *** for p < 0.001)”

The original article has been corrected. The correct presentation of Table 2 is shown below.

**Table 2:** Palindromes within sequences flanking the insertion site. We used the paldpl program to detect approximate palindromes of at least 3 bp with no more than 30% mismatches or indels. This analysis was done in native flanking sequences identified in plant genomes and their randomized (permutated) counterparts, to control for base content effects. We carried out a paired t-test for difference in calculated stem lengths of the native and randomized palindromes. Significant values after Benjamini-Hochberg correction for multiple family testing are marked with an asterisk and printed in bold (* for *p* < 0.1, ** for *p* < 0.01 and *** for *p* < 0.001). Three families with increased mean palindrome stem length after randomization are marked with a tilde

Group	Count	Palindrome length		Paired t-test *p*-value
		*native*	*random*	
ALL	14,813	5.5	5.4	0.000004***
*nested*	*830*	*5.2*	*5.3*	*0.50~*
*non-nested*	*13,983*	*5.5*	*5.4*	0.000001***
Ale	1314	5.5	5.5	0.93
Alesia	21	5.8	5.7	0.75
Angela	91	5.3	5.3	0.93
Athila	1088	5.5	5.3	0.008**
*Bianca*	*443*	*6.0*	*6.1*	*0.97~*
*Bryco*	*29*	*5.8*	*5.9*	*0.95~*
CRM	482	5.3	5.2	0.53
Galadriel	49	5.4	5.1	0.40
Ikeros	348	5.5	5.3	0.10
Ivana	1018	5.5	5.3	0.008**
Ogre	1520	5.5	5.4	0.64
Phygy	285	5.3	5.3	0.94
Reina	852	5.4	5.4	0.67
Retand	2078	5.4	5.3	0.37
Sire	1225	5.4	5.2	0.001**
Tcn1	1947	5.5	5.4	0.001**
TAR	477	5.5	5.4	0.14
Tekay	1029	5.4	5.4	0.61
*Tork*	*517*	*5.6*	*5.8*	0.05*~
