# Supplementary material for: Nested plant LTR retrotransposons target specific regions of other elements, while all LTR retrotransposons often target palindromes and nucleosome-occupied regions: in silico study
Source: Mob DNA. 2019 Dec 14;10:50. doi: 10.1186/s13100-019-0186-z (PMC6911290; doi:10.1186/s13100-019-0186-z)
Supplement: Supplementary file 1 — Additional file 1. Chromosomal localization of nested and non-nested LTR retrotransposons in all studied plant species. [file 13100_2019_186_MOESM1_ESM.pdf]

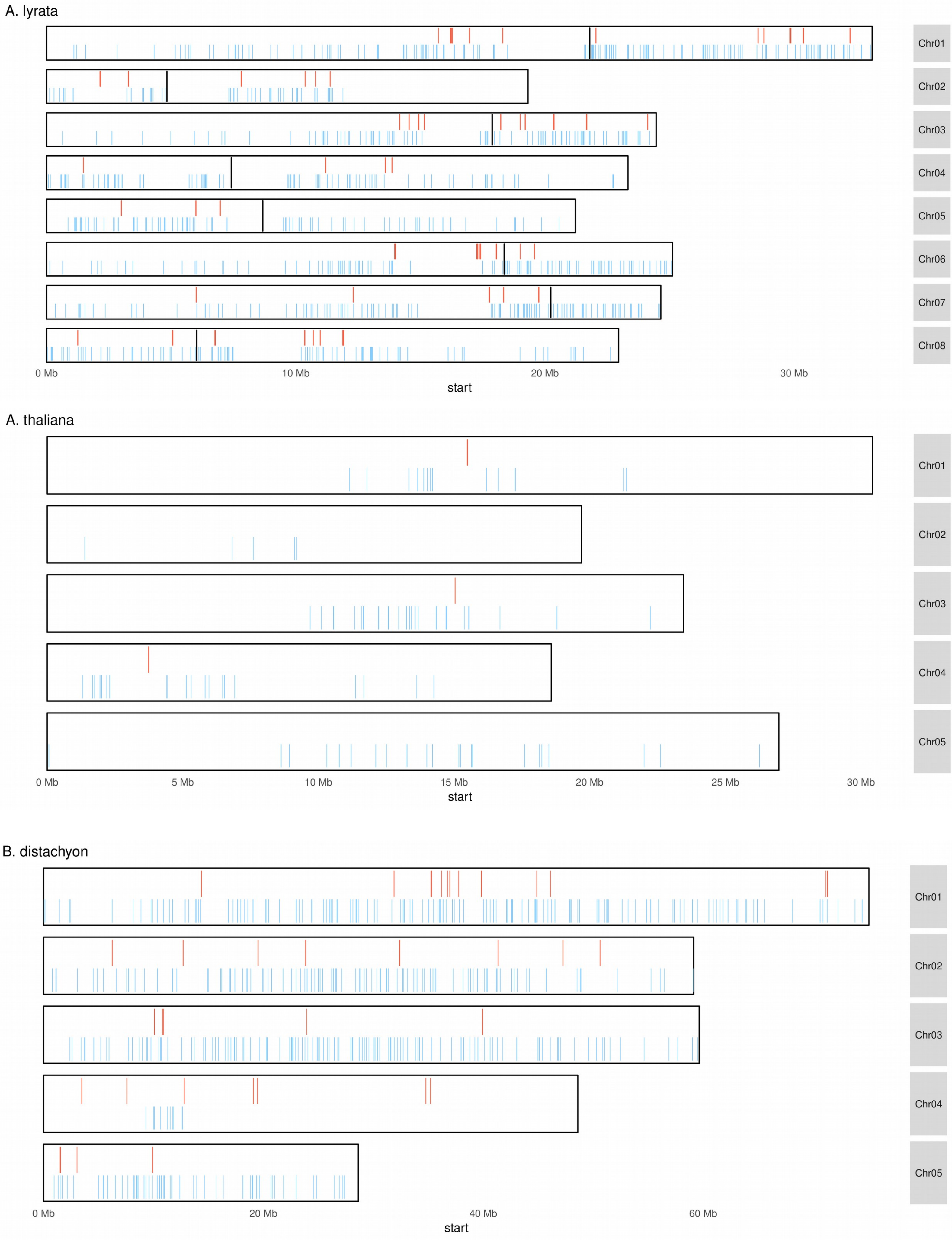

A. lyrata
Chr01
Chr02
Chr03
Chr04
Chr05
Chr06
Chr07
Chr08
0 Mb
10 Mb
20 Mb
30 Mb
start
A. thaliana
Chr01
Chr02
Chr03
Chr04
Chr05
0 Mb
5 Mb
10 Mb
15 Mb
20 Mb
25 Mb
30 Mb
start
B. distachyon
Chr01
Chr02
Chr03
Chr04
Chr05
0 Mb
20 Mb
40 Mb
60 Mb
start

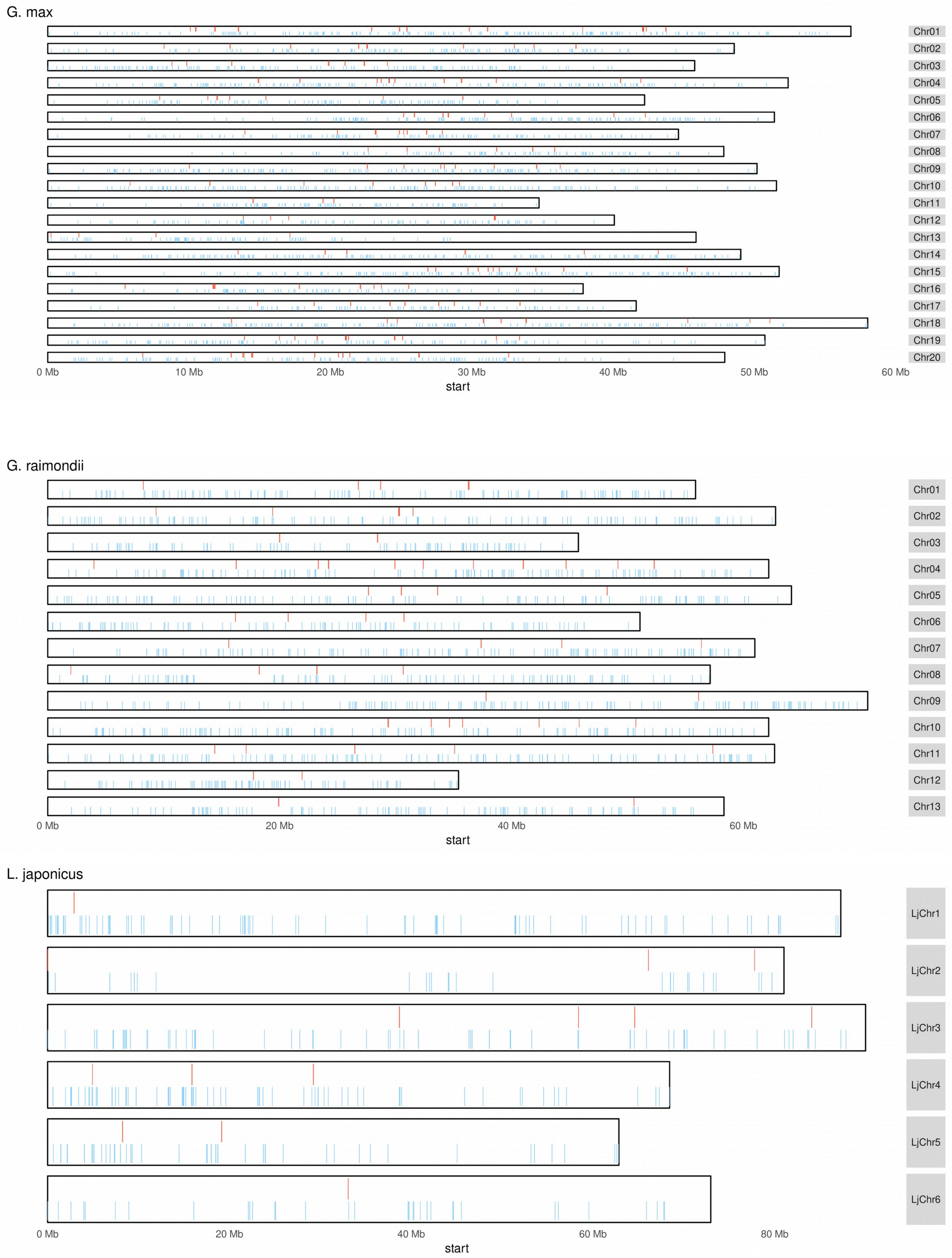
G. max
Chr01
Chr02
Chr03
Chr04
Chr05
Chr06
Chr07
Chr08
Chr09
Chr10
Chr11
Chr12
Chr13
Chr14
Chr15
Chr16
Chr17
Chr18
Chr19
Chr20
0 Mb
10 Mb
20 Mb
30 Mb
40 Mb
50 Mb
60 Mb
start
G. raimondii
Chr01
Chr02
Chr03
Chr04
Chr05
Chr06
Chr07
Chr08
Chr09
Chr10
Chr11
Chr12
Chr13
0 Mb
20 Mb
40 Mb
60 Mb
start
L. japonicus
LjChr1
LjChr2
LjChr3
LjChr4
LjChr5
LjChr6
0 Mb
20 Mb
40 Mb
60 Mb
80 Mb
start

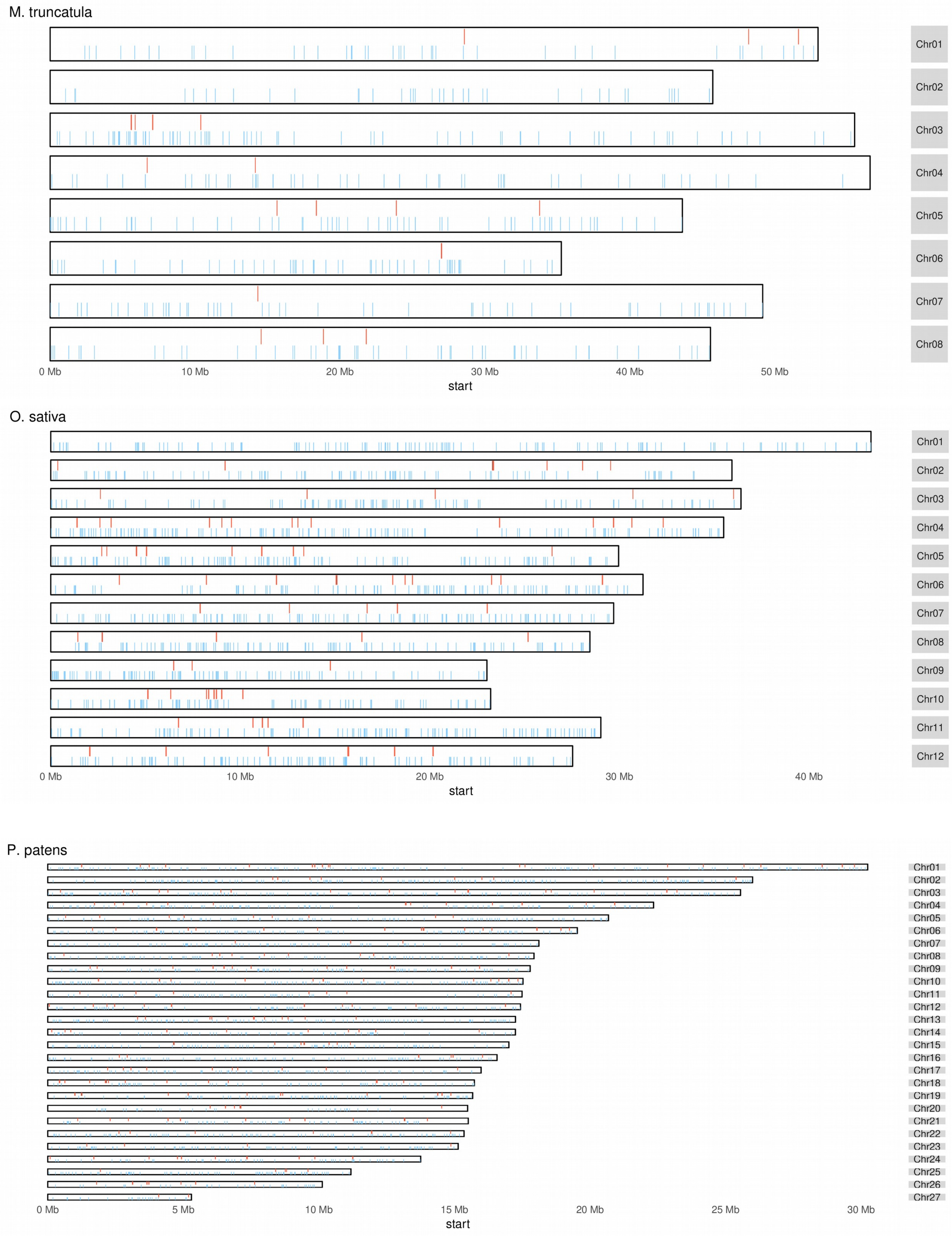
M. truncatula
Chr01
Chr02
Chr03
Chr04
Chr05
Chr06
Chr07
Chr08
0 Mb
10 Mb
20 Mb
30 Mb
40 Mb
50 Mb
start
O. sativa
Chr01
Chr02
Chr03
Chr04
Chr05
Chr06
Chr07
Chr08
Chr09
Chr10
Chr11
Chr12
0 Mb
10 Mb
20 Mb
30 Mb
40 Mb
start
P. patens
Chr01
Chr02
Chr03
Chr04
Chr05
Chr06
Chr07
Chr08
Chr09
Chr10
Chr11
Chr12
Chr13
Chr14
Chr15
Chr16
Chr17
Chr18
Chr19
Chr20
Chr21
Chr22
Chr23
Chr24
Chr25
Chr26
Chr27
0 Mb
5 Mb
10 Mb
15 Mb
20 Mb
25 Mb
30 Mb
start

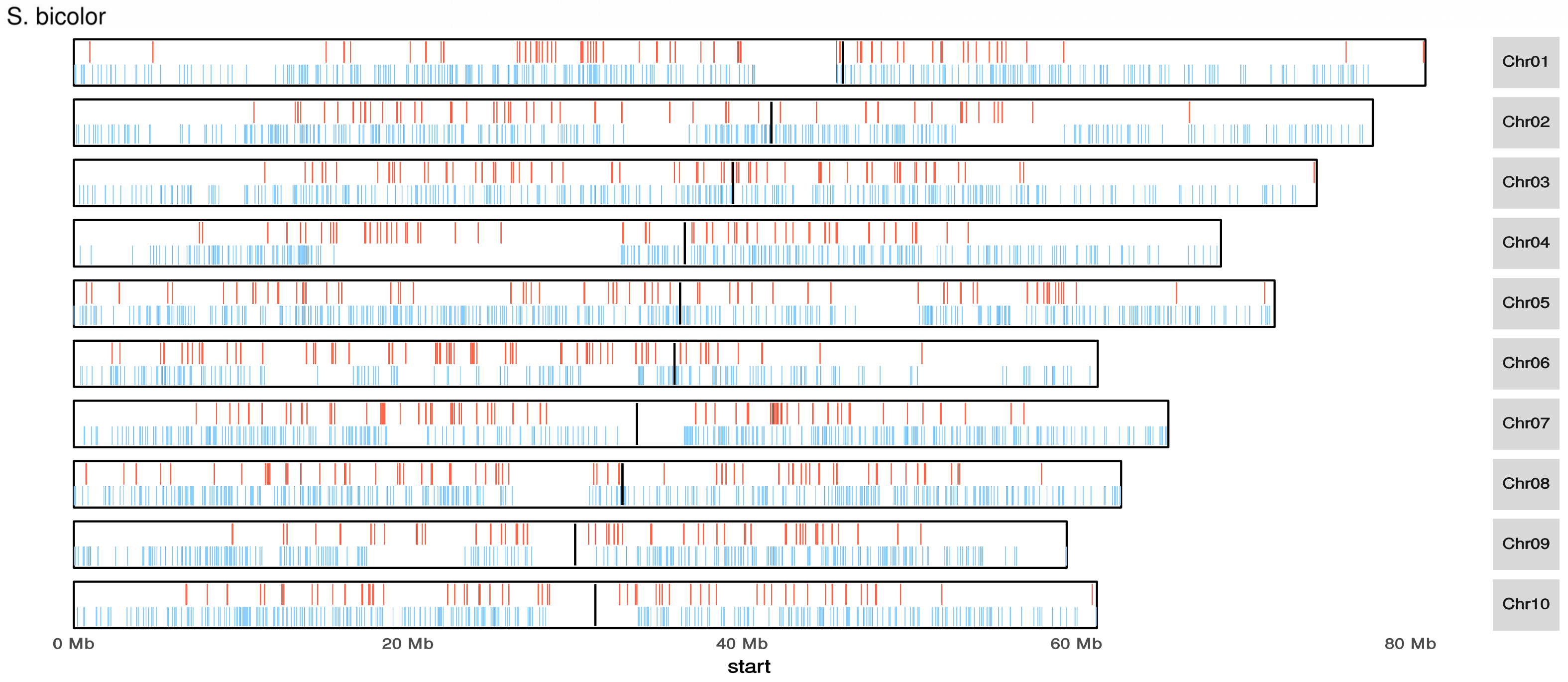
S. bicolor
Chr01
Chr02
Chr03
Chr04
Chr05
Chr06
Chr07
Chr08
Chr09
Chr10
0 Mb
20 Mb
40 Mb
60 Mb
80 Mb
start

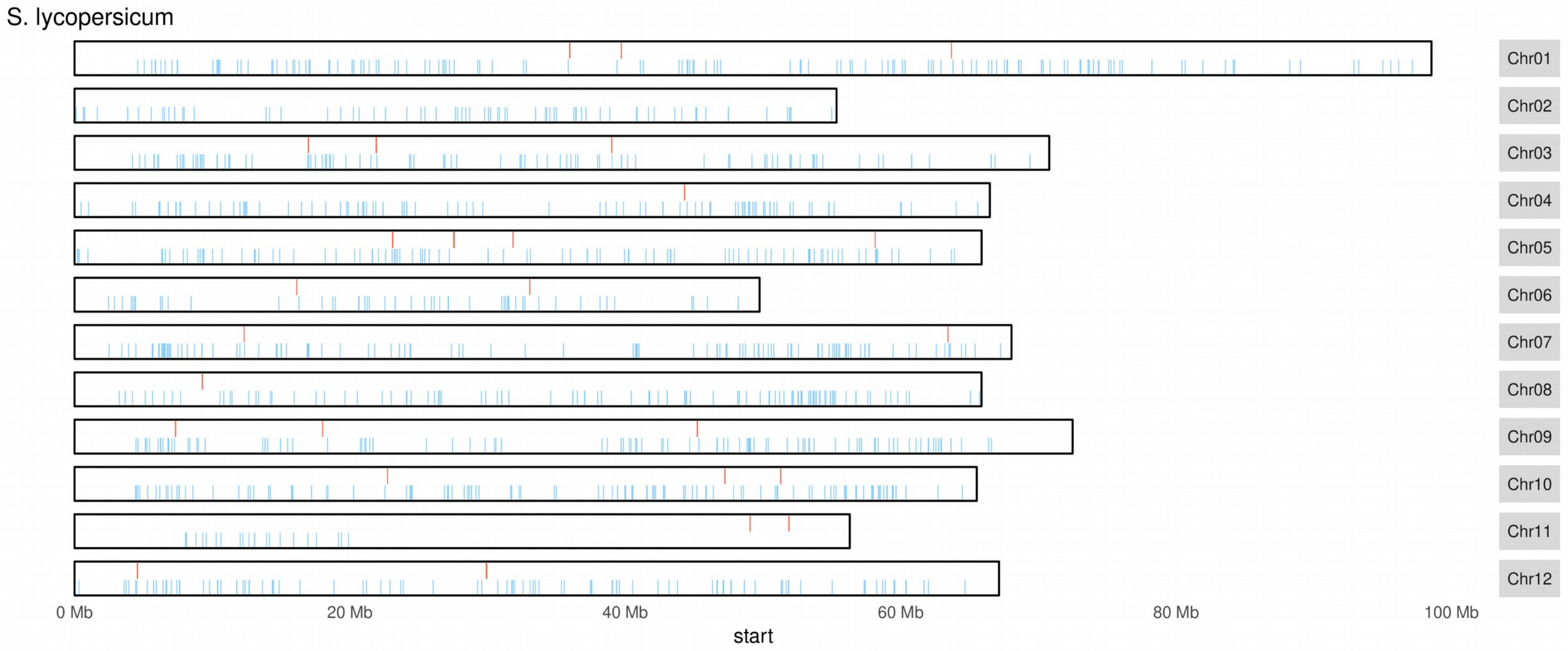
S. lycopersicum
Chr01
Chr02
Chr03
Chr04
Chr05
Chr06
Chr07
Chr08
Chr09
Chr10
Chr11
Chr12
0 Mb
20 Mb
40 Mb
60 Mb
80 Mb
100 Mb
start

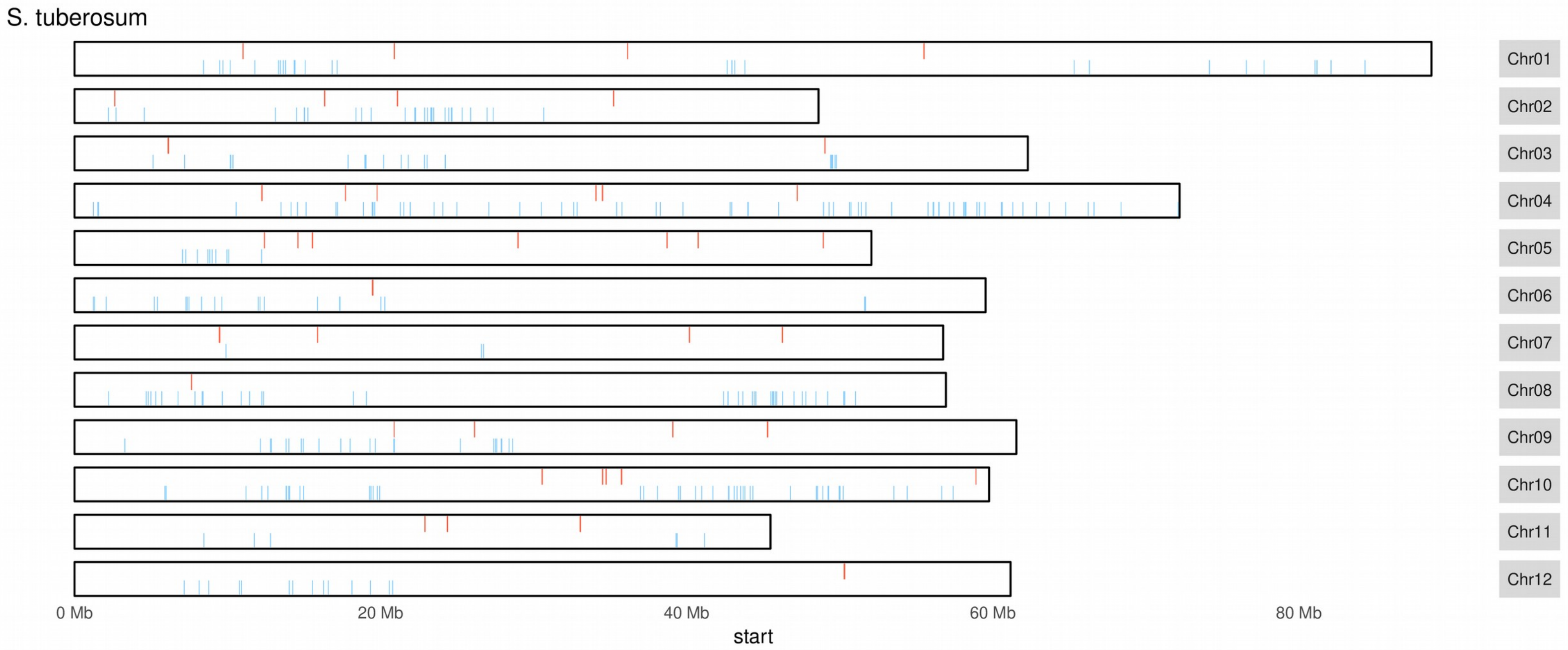
S. tuberosum
Chr01
Chr02
Chr03
Chr04
Chr05
Chr06
Chr07
Chr08
Chr09
Chr10
Chr11
Chr12
0 Mb
20 Mb
40 Mb
60 Mb
80 Mb
start
