# Supplementary material for: Nested plant LTR retrotransposons target specific regions of other elements, while all LTR retrotransposons often target palindromes and nucleosome-occupied regions: in silico study
Source: Mob DNA. 2019 Dec 14;10:50. doi: 10.1186/s13100-019-0186-z (PMC6911290; doi:10.1186/s13100-019-0186-z)
Supplement: Supplementary file 2 — Additional file 2. Tables of statistical support for the preferential nesting into specific regions of LTR retrotransposons (presented in Fig. 3). The number of observed LTR retrotransposon insertion was compared with their expected number normalized by region length. FDR corrected p-values present the results of pairwise comparison after a global chi-squared goodness of fit test. The p-values lower than 0.05 are in bold. [file 13100_2019_186_MOESM2_ESM.pdf]

Additional file 4. Tables of statistical support for the preferential nesting into specific regions of LTR retrotransposons (presented in Fig.3). The number of observed LTR retrotransposon insertion was compared with their expected number normalized by region length. FDR corrected p-values present the results of pairwise comparison after a global chi-squared goodness of fit test. The p-values lower than 0.05 are in bold.

Figure 3A

Ty3/gypsy – all

Region	Observed	Expected	p-value*
LTR left	25.0	60.0	**0.000233**
LTR left - *pbs*	4.0	0.0	0.208823
pbs	0.0	0.0	1.000000
pbs – GAG	126.0	120.1	0.734453
GAG	26.0	39.9	0.104675
GAG – AP	114.0	80.8	**0.022776**
AP	12.0	16.7	0.418465
AP – RT	71.0	55.9	0.208823
RT	49.0	49.4	0.982114
RT – RH	96.0	23.2	**0.000000**
RH	29.0	26.9	0.809135
RH – INT	60.0	78.8	0.130519
INT	27.0	51.3	**0.008096**
INT – CHR	16.0	6.4	0.055613
CHR	3.0	3.0	1.000000
CHR- *ppt*	334.0	271.0	**0.013861**
ppt	0.0	0.0	1.000000
ppt – LTR right	1.0	0.2	0.492865
LTR right	22.0	60.1	**0.000045**

Figure 3B

Ty3/copia – all

Region	Observed	Expected	p-value*
LTR left	3.0	13.1	**0.021834**
LTR left - *pbs*	1.0	0.1	0.451187
pbs	0.0	0.0	1.000000
pbs – GAG	15.0	9.6	0.366352
GAG	10.0	11.5	0.826398
GAG – AP	39.0	28.5	0.267970
AP	3.0	3.1	1.000000
AP – INT	19.0	5.9	**0.016093**
INT	14.0	12.6	0.855640
INT – RT	33.0	27.5	0.567817
RT	12.0	12.5	0.964064
RT – RH	29.0	11.2	**0.009998**
RH	1.0	0.9	0.964064
RH - *ppt*	45.0	43.1	0.901110
ppt	0.0	0.0	1.000000
ppt – LTR right	0.0	0.0	1.000000
LTR right	6.0	14.0	0.109060

Figure 3C

Ty3/gypsy – recent

Region	Observed	Expected	p-value*
LTR left	2.0	4.6	0.410850
LTR left - *pbs*	3.0	0.1	0.160972
pbs	0.0	0.0	1.000000
pbs – GAG	16.0	18.0	0.819570
GAG	4.0	4.3	0.961779
GAG – AP	27.0	14.7	0.103267
AP	1.0	1.4	0.866135
AP – RT	16.0	9.5	0.284381
RT	11.0	8.2	0.623097
RT – RH	18.0	3.8	**0.007041**
RH	2.0	0.7	0.546124
RH – INT	13.0	12.0	0.902403
INT	4.0	7.3	0.421816
INT – CHR	2.0	0.5	0.436054
CHR	0.0	0.0	1.000000
CHR- *ppt*	68.0	51.9	0.223615
ppt	0.0	0.0	1.000000
ppt – LTR right	0.0	0.0	1.000000
LTR right	2.0	2.6	0.856787

Figure 3D

Ty3/copia – recent

Region	Observed	Expected	p-value*
LTR left	0.0	0.0	1.000000
LTR left - *pbs*	0.0	0.0	1.000000
pbs	0.0	0.0	1.000000
pbs – GAG	2.0	0.4	0.525227
GAG	4.0	1.2	0.492007
GAG – AP	11.0	4.7	0.379212
AP	1.0	0.2	0.690779
AP – INT	3.0	0.6	0.483410
INT	3.0	1.1	0.589727
INT – RT	6.0	3.6	0.658505
RT	2.0	1.6	0.971578
RT – RH	4.0	1.1	0.483410
RH	0.0	0.0	1.000000
RH - *ppt*	3.0	6.3	0.525227
ppt	0.0	0.0	1.000000
ppt – LTR right	0.0	0.0	1.000000
LTR right	1.0	2.1	0.744511

Figure 3E

Ty3/gypsy – old

Region	Observed	Expected	p-value*
LTR left	7.0	6.8	1.000000
LTR left - *pbs*	0.0	0.0	1.000000
pbs	0.0	0.0	1.000000
pbs – GAG	19.0	14.6	0.562305
GAG	2.0	3.2	0.709791
GAG – AP	17.0	10.2	0.304034
AP	3.0	1.2	0.513355
AP – RT	6.0	6.5	0.970656
RT	9.0	6.6	0.648118
RT – RH	9.0	1.9	0.087017
RH	5.0	2.9	0.565140
RH – INT	9.0	5.3	0.453029
INT	4.0	4.0	1.000000
INT – CHR	3.0	1.1	0.471661
CHR	1.0	0.5	0.794623
CHR- *ppt*	36.0	32.3	0.756387
ppt	0.0	0.0	1.000000
ppt – LTR right	0.0	0.0	1.000000
LTR right	3.0	3.1	1.000000

* False Discovery Rate corrected p-values

Figure 3F

Ty3/copia – old

Region	Observed	Expected	p-value*
LTR left	1.0	0.5	0.895537
LTR left - *pbs*	0.0	0.0	1.000000
pbs	0.0	0.0	1.000000
pbs – GAG	5.0	2.3	0.550698
GAG	0.0	0.0	1.000000
GAG – AP	4.0	2.8	0.818811
AP	0.0	0.0	1.000000
AP – INT	6.0	0.5	0.217765
INT	3.0	1.6	0.684707
INT – RT	5.0	3.0	0.662076
RT	1.0	0.6	0.941095
RT – RH	5.0	1.4	0.426388
RH	1.0	0.6	0.895537
RH - *ppt*	8.0	5.7	0.710107
ppt	0.0	0.0	1.000000
ppt – LTR right	0.0	0.0	1.000000
LTR right	2.0	1.8	1.000000
