# Supplementary material for: Nested plant LTR retrotransposons target specific regions of other elements, while all LTR retrotransposons often target palindromes and nucleosome-occupied regions: in silico study
Source: Mob DNA. 2019 Dec 14;10:50. doi: 10.1186/s13100-019-0186-z (PMC6911290; doi:10.1186/s13100-019-0186-z)

A

Species	Nested->Original [family]	Number*
P. patens	Phygy->Tcn1	14
P. patens	Tcn1->Tcn1	6
S. bicolor	Retand->Athila	2
S. bicolor	Retand->Retand	1
G. max	SIRE->SIRE	1
G. max	SIRE->Athila	1
G. max	SIRE->Ogre	1

* Number of two nested-original retroelements complexes with identity 80 % and higher.

B

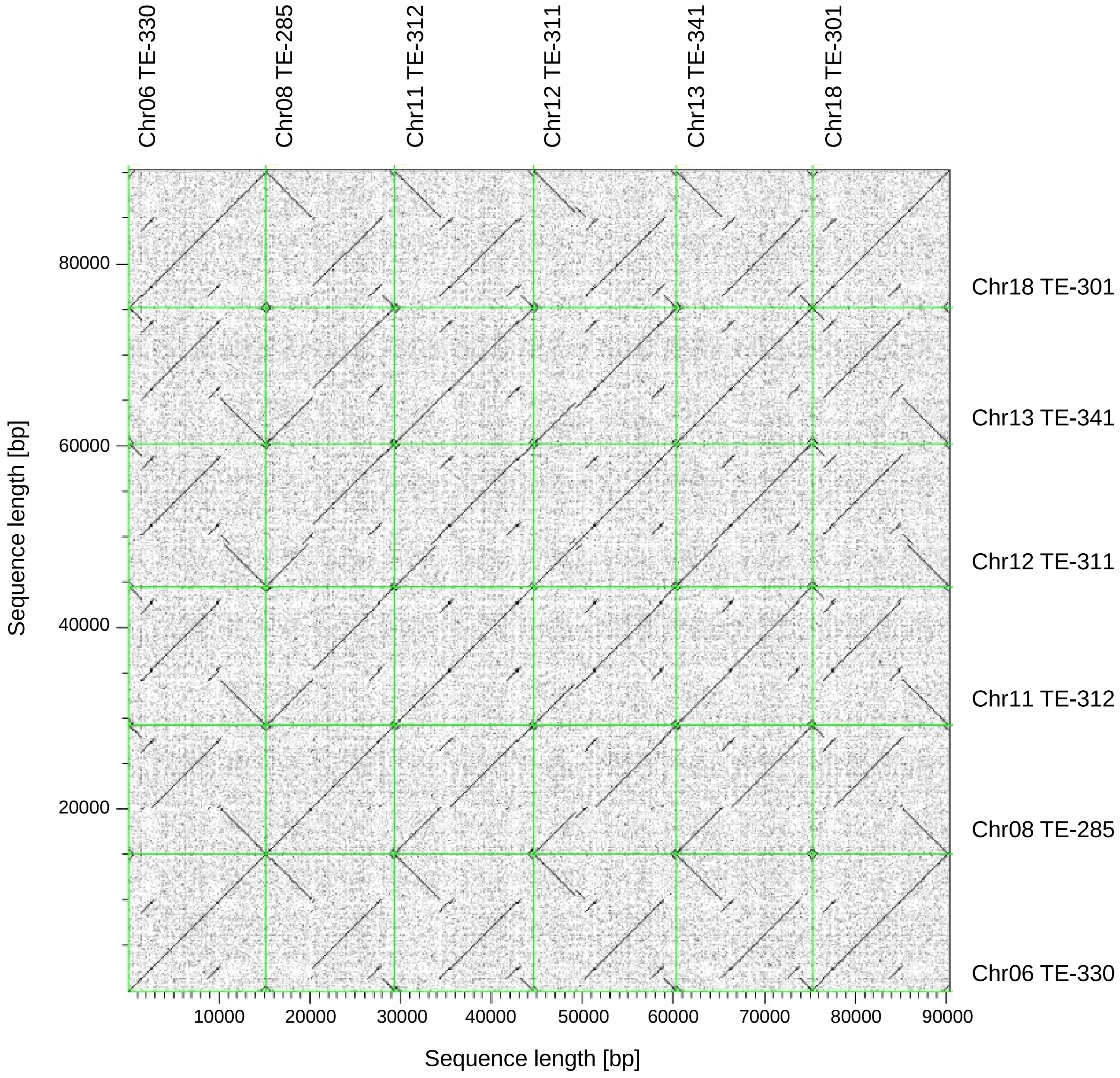

Supplement: Supplementary file 3 — Additional file 3. Copies of nested-original pairs with the high sequence identity. (A) Plant species and LTR retrotransposon family affiliance. (B) Dot plots of P. patens Phygy-Tcn1 complexes with common insertion between INT and CHR. [file 13100_2019_186_MOESM3_ESM.pdf]
