# Supplementary figures and images for: Nested plant LTR retrotransposons target specific regions of other elements, while all LTR retrotransposons often target palindromes and nucleosome-occupied regions: in silico study
Source: Mob DNA. 2019 Dec 14;10:50. doi: 10.1186/s13100-019-0186-z (PMC6911290; doi:10.1186/s13100-019-0186-z)

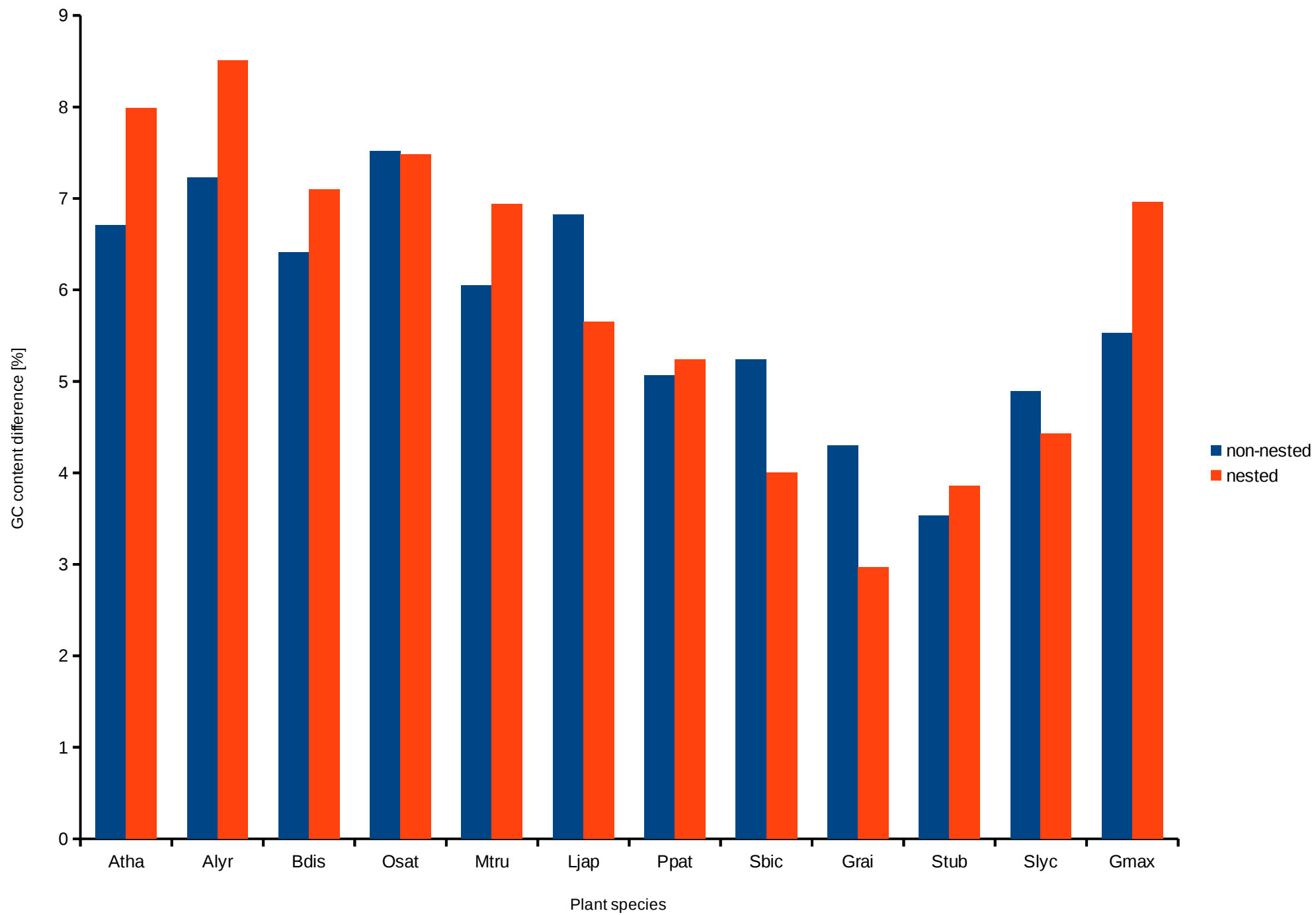

Supplement: Supplementary file 4 — Additional file 4. Difference in GC content between LTR retrotransposons and whole genome sequence in all plant species. [file 13100_2019_186_MOESM4_ESM.pdf]

**A**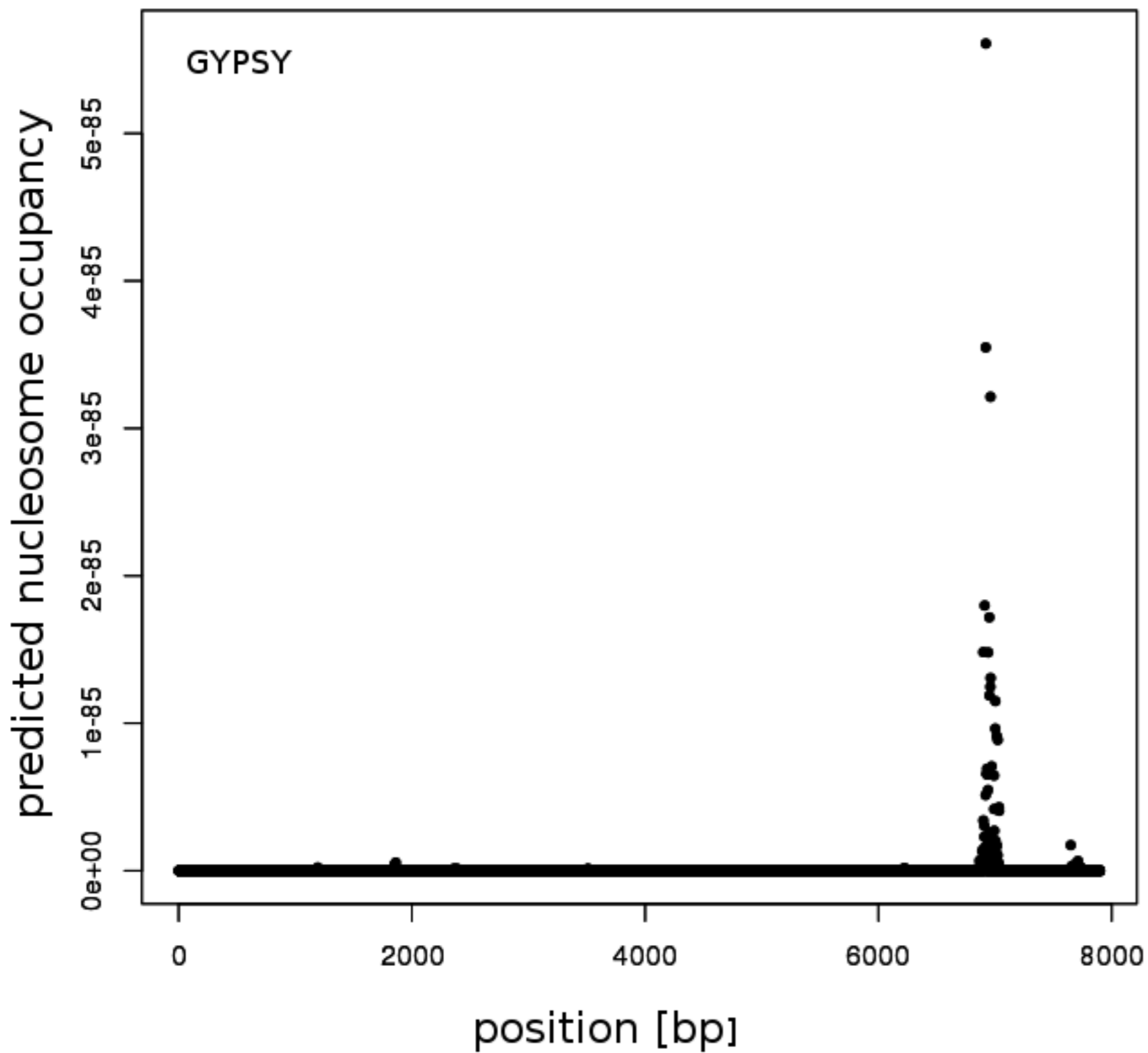**B**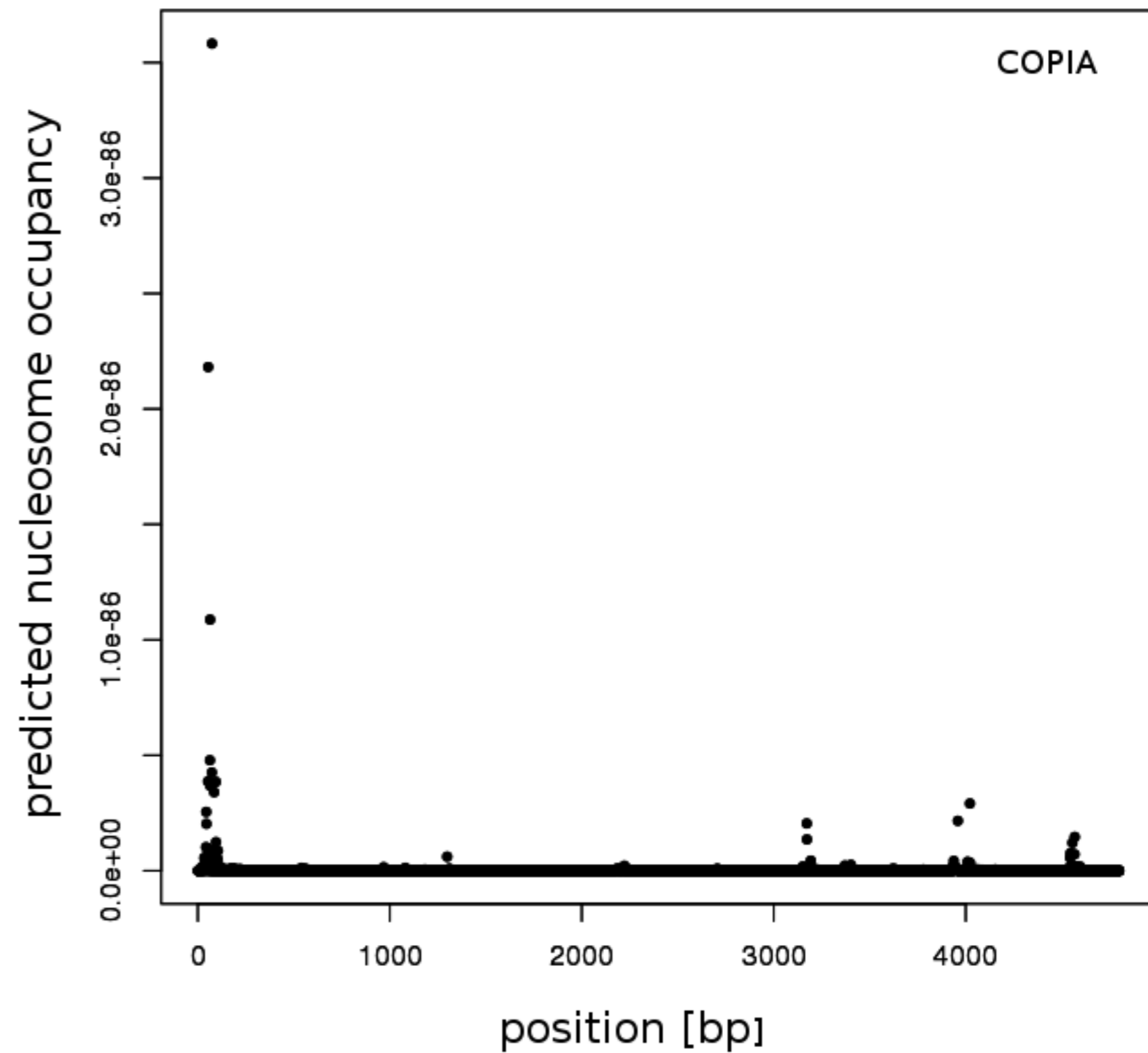

Supplement: Supplementary file 5 — Additional file 5. Nucleosome occupancy prediction counted for Ty3/gypsy or Ty1/copia superfamilies members detected as nested or non-nested LTR retrotransposons in this study. [file 13100_2019_186_MOESM5_ESM.pdf]
